# Altered quorum sensing and physiology of *Staphylococcus aureus* during spaceflight detected by multi-omics data analysis

**DOI:** 10.1038/s41526-023-00343-7

**Published:** 2024-01-08

**Authors:** Matthew R. Hauserman, Mariola J. Ferraro, Ronan K. Carroll, Kelly C. Rice

**Affiliations:** 1 Department of Microbiology and Cell Science, IFAS, University of Florida, Gainesville, FL USA; 2 Department of Biological Sciences, Ohio University, Athens, OH USA

**Keywords:** Microbiology, Molecular biology

## Abstract

*Staphylococcus aureus* colonizes the nares of approximately 30% of humans, a risk factor for opportunistic infections. To gain insight into *S. aureus* virulence potential in the spaceflight environment, we analyzed RNA-Seq, cellular proteomics, and metabolomics data from the “Biological Research in Canisters-23” (BRIC-23) GeneLab spaceflight experiment, a mission designed to measure the response of *S. aureus* to growth in low earth orbit on the international space station. This experiment used Biological Research in Canisters-Petri Dish Fixation Units (BRIC-PDFUs) to grow asynchronous ground control and spaceflight cultures of *S. aureus* for 48 h. RNAIII, the effector of the Accessory Gene Regulator (Agr) quorum sensing system, was the most highly upregulated gene transcript in spaceflight relative to ground controls. The *agr* operon gene transcripts were also highly upregulated during spaceflight, followed by genes encoding phenol-soluble modulins and secreted proteases, which are positively regulated by Agr. Upregulated spaceflight genes/proteins also had functions related to urease activity, type VII-like Ess secretion, and copper transport. We also performed secretome analysis of BRIC-23 culture supernatants, which revealed that spaceflight samples had increased abundance of secreted virulence factors, including Agr-regulated proteases (SspA, SspB), staphylococcal nuclease (Nuc), and EsxA (secreted by the Ess system). These data also indicated that *S. aureus* metabolism is altered in spaceflight conditions relative to the ground controls. Collectively, these data suggest that *S. aureus* experiences increased quorum sensing and altered expression of virulence factors in response to the spaceflight environment that may impact its pathogenic potential.

## Introduction

*Staphylococcus aureus* is a pernicious pathogen capable of infecting nearly every tissue and organ system in the human body. Most *S. aureus* strains are resistant to multiple antibiotics, and certain isolates are resistant to nearly all antibiotics used in the clinical setting. The CDC 2019 Antibiotic Threats Report listed methicillin-resistant *S. aureus* (MRSA) at a “serious” threat level, with over 10,000 estimated deaths in 2017 alone^1^. *S. aureus* is a frequent cause of hospital-acquired infections, and in recent years, highly virulent and transmissible community-acquired methicillin-resistant *S. aureus* (CA-MRSA) strains have emerged. Approximately 30% of humans are nasally colonized by *S. aureus*, which represents a significant risk factor for invasive infections^2^. As a pathogen, *S. aureus* expresses a vast repertoire of cell surface adhesins, secreted toxins, and tissue-degrading enzymes that contribute to its ability to colonize host tissue, evade the immune system, and transition from a localized to systemic infection. The expression of these virulence factors is coordinated by a complex network of genetic regulators, including the Accessory Gene Regulator (Agr) quorum-sensing system, which has been extensively reviewed^3–7^. The *agr* operon is comprised of two transcripts, RNAII and RNAIII, which are activated by the P2 and P3 promoters, respectively^8^. RNAIII is an untranslated RNA species that regulates a variety of target genes at both the transcriptional and post-transcriptional level, making it a primary downstream effector of the Agr system, whereas the RNAII transcript contains the *agrBDCA* genes [reviewed in refs. ^6,7^]: AgrD, which is processed and secreted out of the cell in a form known as the autoinducing peptide (AIP), AgrB, the membrane protein that performs this processing and secretion, AgrC, a sensor kinase which recognizes the AIP and transduces the signal to AgrA, the response regulator which activates transcription at the P2 and P3 promoters.

*S. aureus* also poses a potential health risk to astronauts during long-term spaceflight missions. This bacterium was recovered from the nasal passages of Apollo 13 and 14 astronauts at significantly higher numbers compared to preflight samples collected from the same astronauts^9^, and *S. aureus* was also among the most frequently isolated bacteria from astronauts that participated in 25 Space Shuttle missions (STS-26 to STS-50)^10^. Furthermore, several studies monitoring the presence of microbes aboard the International Space Station (ISS)^11–13^ as well as in the environment of a full-duration simulation of a crewed return flight to Mars^14^ identified staphylococci as being predominant. Because of the potential threat of *S. aureus* to astronaut health, this pathogen’s stress resistance and virulence potential have been studied in both a spaceflight experiment^15^ and in ground-based models^15–19^ of simulated microgravity. In the previous spaceflight experiment, *S. aureus* virulence was reduced when assessed as a function of the quantity of residual bacteria in the presence and absence of *Caenorhabditis elegans*^15^. However, these studies were conducted using a nematode feeding model, which does not entirely reflect the complexity of *S. aureus* pathogenesis in humans.

NASA previously conducted a “Biological Research in Canisters-23” (BRIC-23) spaceflight experiment (OSDR accession# OSD-145), designed to measure the response of both *Bacillus subtilis* and *S. aureus* to the spaceflight environment^20,21^. In this experiment, Biological Research in Canisters-Petri Dish Fixation Units (BRIC-PDFUs)^22^ were used to grow asynchronous ground controls and spaceflight samples of *S. aureus* strain UAMS-1, a clinical MSSA isolate that was originally isolated from an osteomyelitis infection^23^. Herein, our analysis of the *S. aureus* RNA-Seq, proteomics, and metabolomics datasets from BRIC-23 suggests that *S. aureus* spaceflight samples may have altered metabolism (increased amino acid metabolism, TCA cycle, and transport of non-preferred carbon sources, and decreased glycolysis/fermentation and translation machinery) relative to ground-control cultures. Furthermore, *agr* genes and several positively regulated targets were the most highly upregulated transcripts in spaceflight. We also performed secretome analysis of culture supernatants from the BRIC-23 spaceflight and ground control cultures, in which several Agr-regulated secreted proteins were increased in abundance in the spaceflight samples. Collectively, these data suggest that *S. aureus* experiences altered metabolism, increased quorum sensing, and altered expression of virulence factors in response to the spaceflight environment that may impact its pathogenic potential.

## Results

### Experimental considerations, sample variability, and clustering amongst FLT and GC samples

**Fig. 1:**
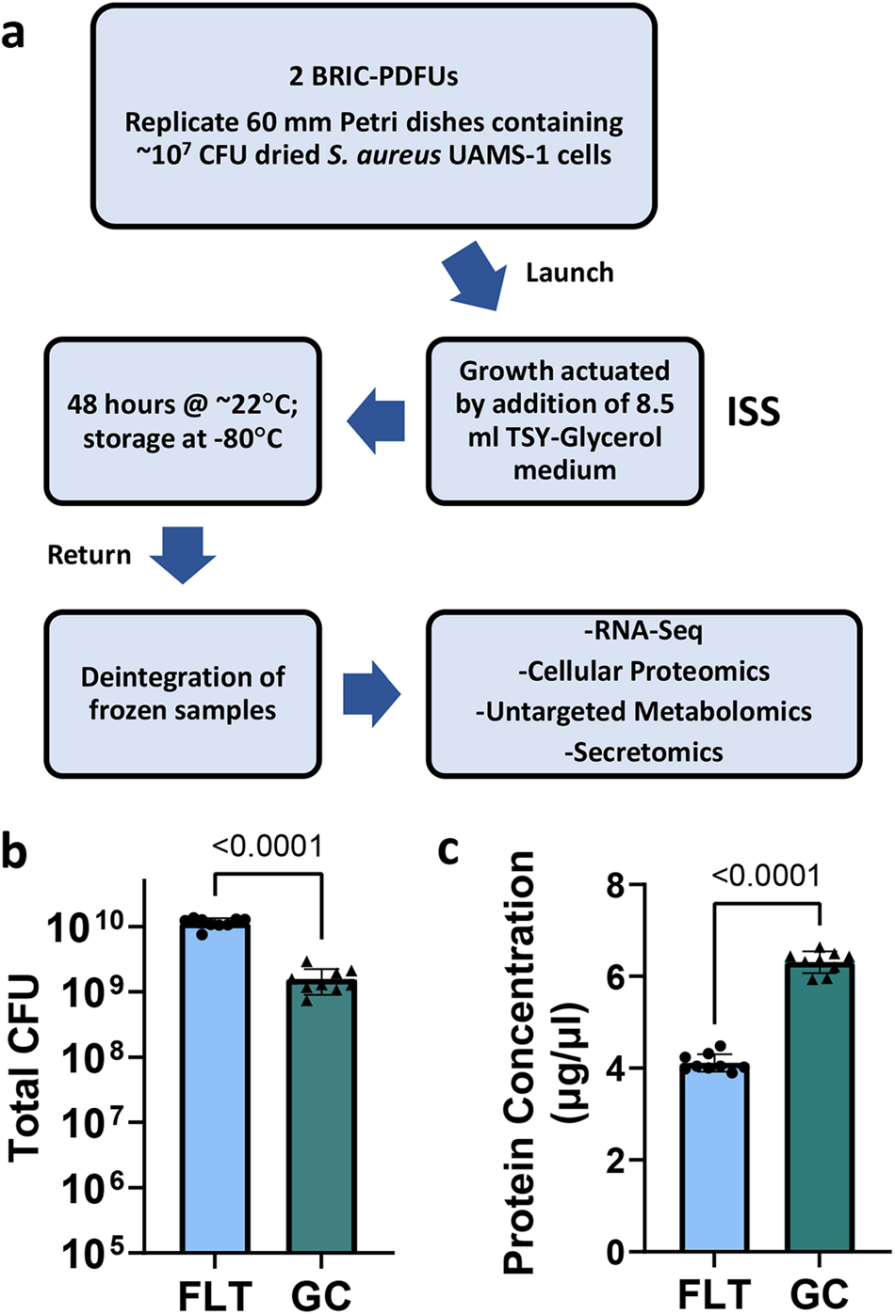
BRIC-23 Flight Experiment Overview and Associated Data. **a** Overview of BRIC-23 Flight Experiment. Full details have been previously published in^20,21^. **b** Average total CFU harvested from FLT and GC cultures. **c** Average protein concentration (measured after concentration and removal of < 5 kDa peptides/amino acids) of culture supernatants from FLT and GC cultures. For B and C, data represent the average of *n* = 9 biological samples per growth condition, error bars = standard deviation. *P*-values (two-tailed t-test) are shown.

As outlined in Fig. 1a, the BRIC-23 flight (FLT) experiment and corresponding asynchronous ground controls (GCs) were conducted on *S. aureus* cultures grown to a single time point (48 h) at ISS ambient ( ~ 22 °C) temperature. The total CFUs per petri dish harvested from both flight and ground control experiments (as reported in OSDR entry OSD-145) suggested that the FLT cultures may have grown to higher cell densities compared to GC cultures (Fig. 1b).

**Fig. 2:**
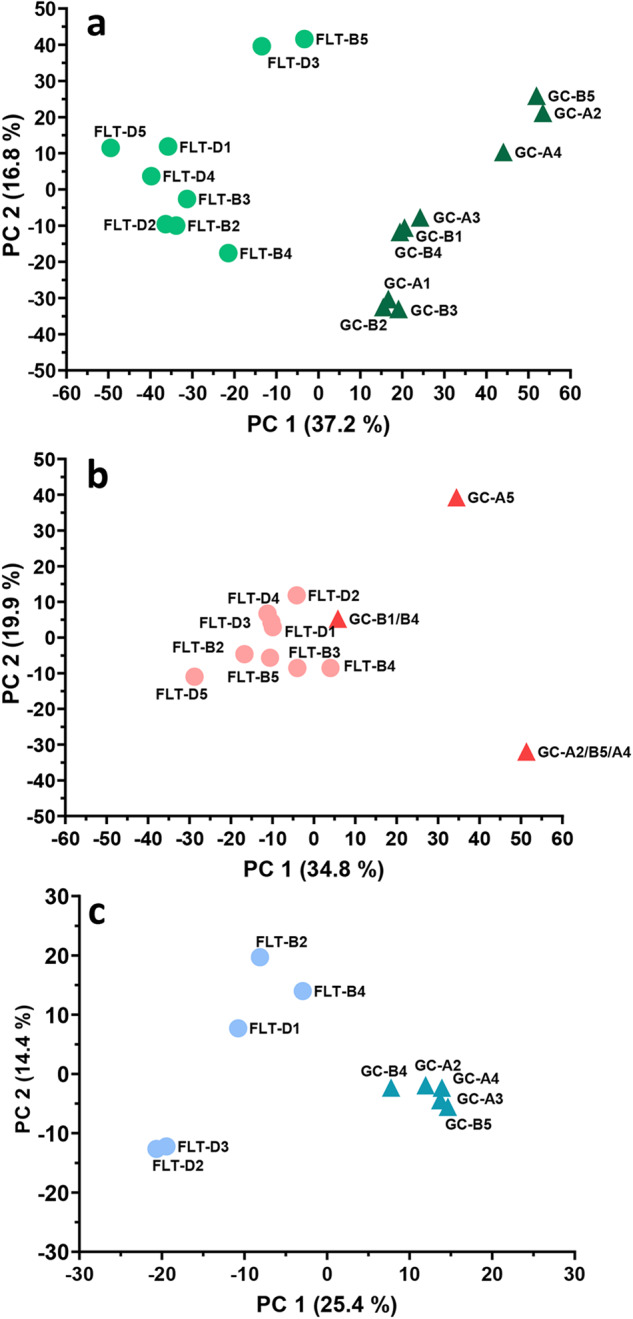
Principal Component Analysis (PCA) of Omics Data. **a** RNA-Seq (*n* = 9 biological samples each for FLT and GC). **b** Proteomics (*n* = 9 FLT and *n* = 3 GC biological samples). **c** Secretomics (*n* = 5 biological samples each for FLT and GC). In all graphs, BRIC-23 flight (FLT; circles) and ground control (GC; triangles) samples are indicated. PCA analysis of RNA-Seq data was performed on normalized RPKM values for all genes using CLC genomics workbench, and PCA analysis of proteomics and secretomics was performed on scaled abundances and normalized weighted spectra, respectively, using Clustvis^89^.

Principle components analysis (PCA) was performed on all quantile normalized Reads Per Kilobase of transcript, per Million mapped reads (RPKM) gene expression values from the BRIC-23 FLT and GC RNA-Seq samples (*n* = 9 per experimental group) (Fig. 2a). This analysis revealed that the samples within each experimental group showed robust clustering, and that most of the sample variability correlated with experimental group (FLT vs. GC) along the PC1 axis. Cellular proteomics (Fig. 2b) and secretomics (Fig. 2c) PCA of scaled abundances and normalized weighted spectra, respectively, revealed similar trends as the RNA-Seq data. All datasets were also subjected to differential expression/abundance analysis to identify differences in transcript and protein relative abundances between spaceflight and ground control samples. FLT and GC genes that showed statistically significant differences in relative abundance ( > 2-fold change) in the RNA-Seq dataset (summarized in Supplemental Table 1) were also subjected to hierarchical clustering analysis, which showed robust clustering amongst the biological replicates within each experimental group (*n* = 9 each), indicating consistency of the data across replicates in each group (Supplemental Fig. 1). For cellular proteomics, the metadata associated with this experiment in GeneLab indicated that due to low protein yield from some of the GC samples, replicates had to be pooled prior to proteomics, resulting in only *n* = 3 GC samples and *n* = 9 FLT samples. However, hierarchical clustering analysis on the FLT and GC proteins that showed statistically significant differences in relative abundance ( > 1.5-fold change, Supplemental Table 2) indicated good consistency and clustering between the biological replicates within each experimental group (Supplemental Fig. 1). FLT and GC samples (*n* = 5 each) analyzed for secretomics also displayed strong clustering among proteins with significant differences in relative abundance ( > 1.5-fold change, Supplemental Fig. 1 and Supplemental Table 3). Interestingly, the average concentration of secreted proteins of FLT sample supernatants was approximately 30% less than that of the GC culture supernatants (Fig. 1c; measured after concentration and removal of < 5 kDa peptides/amino acids).

### Overlap between RNA-Seq, proteomics, and secretomics datasets

**Fig. 3:**
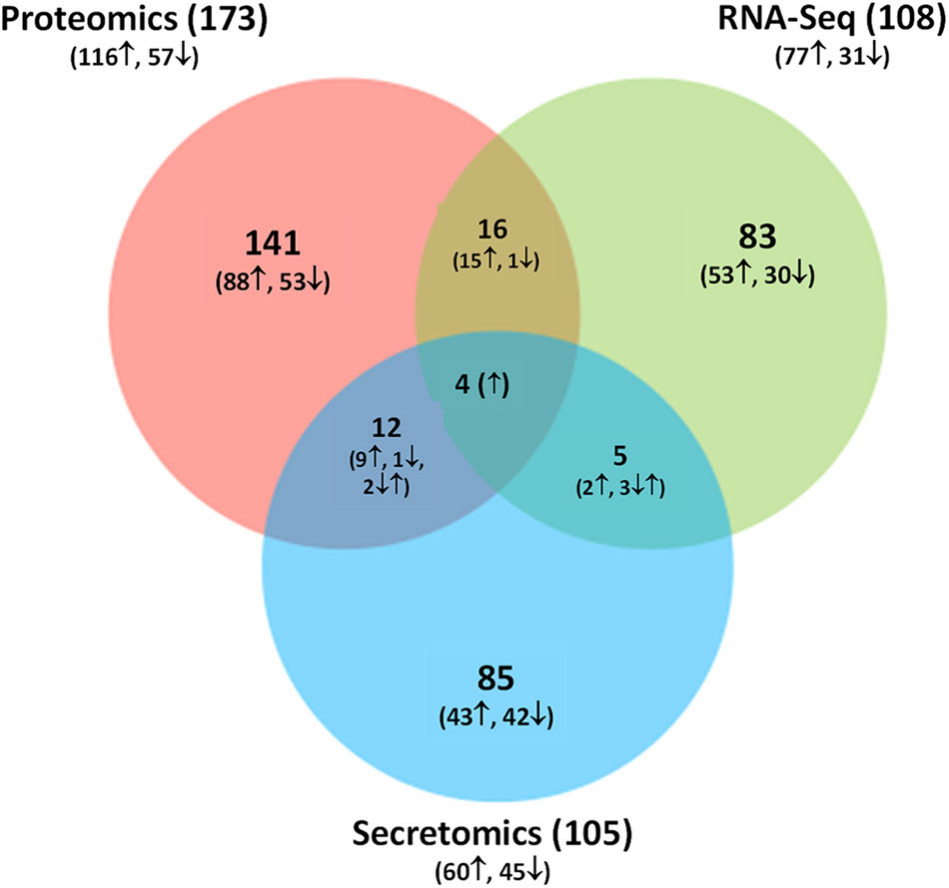
Venn diagram showing the number of DE genes/proteins in RNA-Seq, cellular proteomics, and secretomics BRIC-23 datasets. BRIC-23 RNA-Seq data cutoffs: DE fold-change ≥ 2, *P*-value < 0.000016 (2-tailed t-test), and mean normalized expression value ≥ 10 for both samples. BRIC-23 cellular proteomics and secretomics data cutoffs: *P*-value < 0.05 (Student t-test), fold-change > 1.5.

In total, 386 genes and proteins were identified across the RNA-Seq, cellular proteomics, and secretomics datasets that exceeded the respective cutoff values of 2-fold for RNA-Seq and 1.5-fold for proteomics (Fig. 3), with 37 genes/proteins that were common to at least two datasets (Table 1). Of the twenty genes that were common to RNA-Seq and cellular proteomics (Table 1), nineteen displayed increased abundance in spaceflight, including multiple genes and proteins related to the urease pathway and Type VII secretion, as well as two genes related to copper transport and two subunits of L-serine dehydratase. Notably, AgrA, the main response regulator of the *agr* quorum sensing system, was found to have both increased transcript levels and increased protein abundance in spaceflight samples. The sole protein downregulated in both datasets was a predicted GMP reductase, responsible for interconversion of purine nucleotides. Eight of the nine genes and proteins overlapping between the RNA-Seq and secretomics datasets were primarily annotated as intracellular proteins, involved in several different pathways. The lone extracellular protein in this dataset, EsxA, is secreted by the Type VII-like Ess secretion system^24^. Other genes and proteins of the Ess-related secretion system (EsaA, EsaB, EssC) were also upregulated in both the RNA-Seq and cellular proteomics datasets (Table 1). Four genes and proteins were common to all three datasets, and all displayed increased abundance in spaceflight in each analysis. These consisted of the urease alpha and beta subunits, the urease accessory protein UreG, and the secreted EsxA protein described above (Table 1).

**Table 1:** Summary of overlapping gene transcripts and proteins identified as statistically significant and meeting fold-change cutoff criteria in RNA-Seq, proteomics, and secretomics DE analyses.

Gene ID	Function	RNA-Seq^a^	Proteomics^a^	Secretomics^a^
	Type VII secretion system			
SAR0279	6 kDa early secretory antigenic target ESAT-6 (EsxA)	4.61	1.61	7.50
SAR0280	Type VII secretion protein EsaA	3.01	2.03	
SAR0283	Type VII secretion protein EssB	4.28	2.55	
SAR0284	Type VII secretion system protein EssC	4.73	1.71	
	Agr-regulated			
SAR2126	Response regulator transcription factor AgrA	14.30	3.96	
SAR1021	Cysteine protease precursor; SspB		2.55	13.07
SAR1022	Glutamyl endopeptidase precursor; V8 Protease; SspA		3.48	6.95
SAR2716	Zinc metalloproteinase aureolysin; Aur		3.27	2.19
	Copper-associated			
SAR2637	Copper-exporting P-type ATPase A (CopA)	2.91	2.55	
SAR2639	CopZ putative heavy-metal-associated protein		3.11	18.19
SAR0720	Copper-translocating P-type ATPase	2.19	2.55	
SAR0721	Multicopper oxidase protein		1.93	4.08
	Arginine and Urea Metabolism			
SAR2714	Arginine deiminase		2.05	3.04
SAR2374	Urease alpha subunit	4.91	2.28	1.82
SAR2373	Urease beta subunit	5.02	2.15	4.35
SAR2375	Urease accessory protein UreE	4.66	1.91	
SAR2376	Urease accessory protein UreF	4.37	2.37	
SAR2377	Urease accessory protein UreG	4.37	1.85	4.37
SAR2378	Urease accessory protein UreD	4.12	2.53	
	Misc. cell processes			
SAR2611	L-serine dehydratase, beta subunit	2.76	2.10	
SAR2610	L-serine dehydratase, alpha subunit	2.19	1.63	
SAR1425	2-oxoglutarate dehydrogenase E1 component	2.03	1.55	
SAR1870	Methionine adenosyltransferase		1.63	1.61
SAR1851	Riboflavin biosynthesis protein RibBA; GTP cyclohydrolase II	2.02	1.73	
SAR2775	2-oxoglutarate/malate translocator-like protein	2.05	1.94	
SAR1984	Bacterial non-heme ferritin	2.13		1.58
SAR0278	Secreted antigen precursor; staphyloxanthin biosynthesis	3.00	2.71	
SAR1347	GMP reductase (predicted)	−2.07	−1.66	
SAR0155	Capsular polysaccharide synthesis enzyme	2.15		−2.56
SAR2359	Putative molybdenum cofactor biosynthesis protein B		−1.91	2.91
SAR1127	Hypothetical protein, similarity with fibrinogen-binding protein Efb	3.78		−2.36
SAR2506	Phosphoglycerate mutase	2.55		−1.56
SAR1797	30 S ribosomal protein S4		−2.10	1.69
	Hypothetical			
SAR0007	ADP-dependent (S)-NAD(P)H-hydrate dehydratase	2.46		1.80
SAR0345	Conserved hypothetical protein		1.52	2.94
SAR2028	Aminotransferase class I/II-fold pyridoxal phosphate-dependent enzyme		1.55	2.03
SAR2054	Hypothetical phage protein		−1.55	−1.53

^a^Numbers represent the fold-Change (FLT/GC). Plus (+) and minus (-) symbols designate upregulated and downregulated, respectively, in FLT samples relative to GC samples.

### Overview of gene and protein functional categories identified in multi-omics analysis

**Fig. 4:**
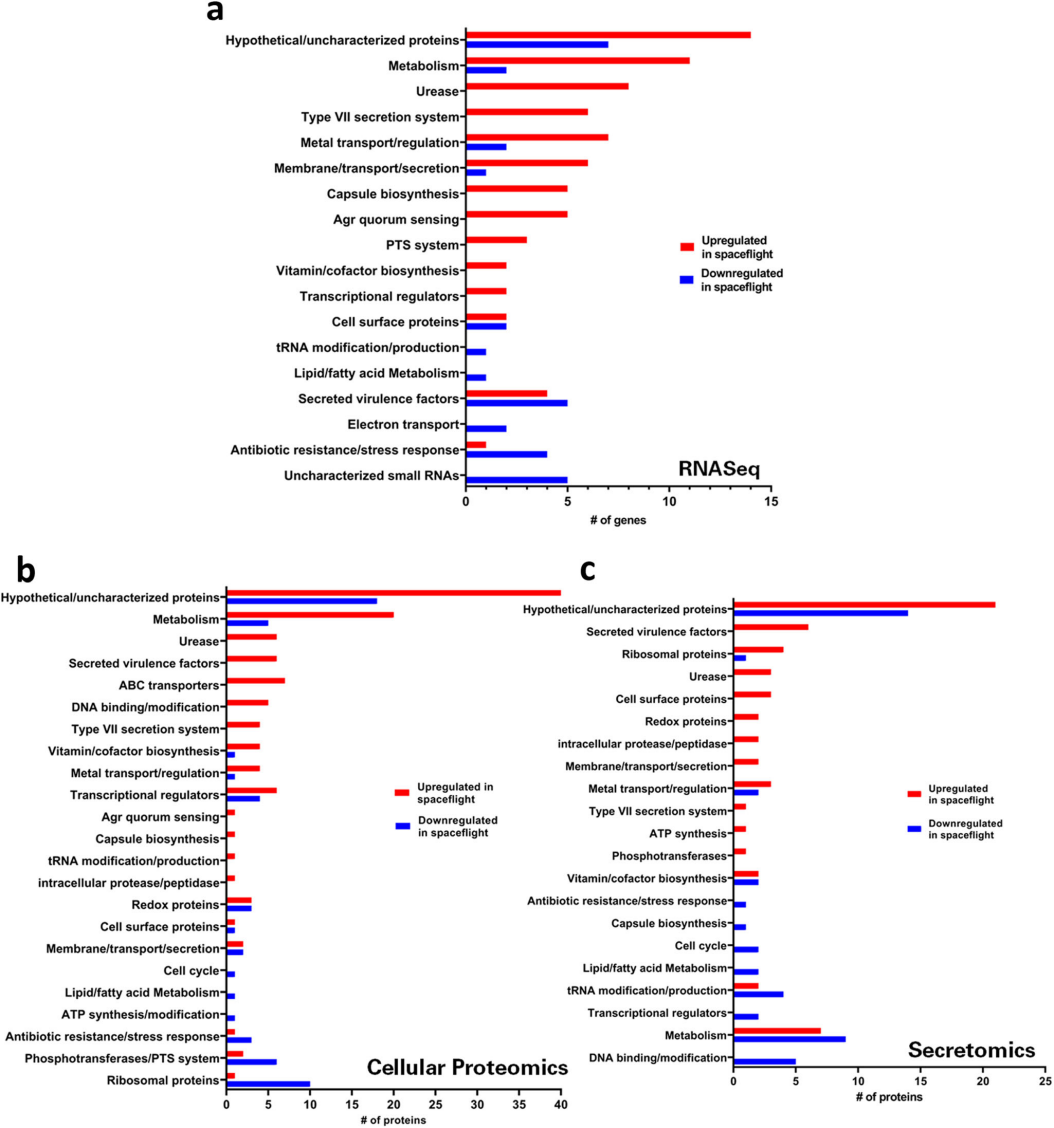
Distribution of *S. aureus* UAMS-1 gene/protein functional categories differentially expressed (DE) during spaceflight relative to ground control. **a** BRIC-23 RNA-Seq data was subjected to DE analysis, with cutoffs (DE fold-change ≥ 2, *P*-value < 0.000016 and mean normalized expression value ≥ 10 for both samples) applied prior to assignment of functional categories (based on *S. aureus* genome annotations and/or UNIProt database). **b**, **c** BRIC-23 cellular proteomics (B) and secretomics (C) functional categories were assigned based on *S. aureus* genome annotations and/or UNIProt database (*P*-value < 0.05, fold-change > 1.5).

The statistically significant changes in transcripts and proteins that fell within the fold-change cutoff criteria for each dataset ( > 2-fold for RNA-Seq, > 1.5-fold for proteomics and secretomics) were manually curated and functionally categorized to broadly examine which aspects of *S. aureus* physiology and virulence were affected by spaceflight conditions (Fig. 4). All datasets contained many hypothetical or uncharacterized proteins and genes, accounting for approximately 21% of all DE genes (RNA-Seq, Fig. 4a), 33% of all DE cellular proteins (proteomics, Fig. 4b), and 33% of all DE secreted proteins (secretomics, Fig. 4c). In the RNA-Seq DE analysis, gene transcripts related to extracellular transport, urease, and Type VII secretion were among the most commonly upregulated, along with genes involved in capsule biosynthesis and metabolism of a variety of substrates, including amino acids and carbohydrates (Fig. 4a). As discussed in more detail below, genes of the *agr* P2 (*agrBDCA*) and P3 (*RNAIII*) transcripts represented the five most highly upregulated transcripts (14 to 88-fold) in FLT samples (Supplemental Table 1). Additionally, expression of many Agr-independent virulence factors was upregulated in spaceflight relative to the ground controls (discussed in more detail below), as well as expression of genes and/or proteins involved in DNA-binding, transcriptional regulators, ABC transporters, urease subunits, and metal acquisition (Fig. 4). Ribosomal proteins was the most commonly downregulated category in FLT samples amongst cellular proteins, followed by those involved in glycolysis, ATP synthesis, and stress resistance. Additionally, a number of small RNAs (RNA-Seq) were downregulated in FLT samples relative to GC samples (Fig. 4a).

Functional enrichment amongst these omics datasets was also assessed by PaintOmics^25^ and STRING^26^ (Table 2 and Supplemental Table 4, respectively), which confirmed the patterns of gene and protein functional categories observed in Fig. 4. Using PaintOmics, an overrepresentation of significant biological features (P < 0.05) of pathways related to arginine biosynthesis, purine metabolism, thiamine metabolism, quorum sensing, and phosphotransferase systems (PTS) were common to both the RNA-Seq and proteomics datasets (Table 2). Additionally, pathways related to ribosome function and fructose/mannose metabolism were significantly enriched in the proteomics dataset, and two-component system and riboflavin metabolism were significantly enriched in the RNA-Seq dataset (Table 2). STRING analysis of RNA-Seq, proteomics, and secretomics DE genes/proteins (meeting statistical and fold-change cutoff criteria) revealed significant (FDR-adjusted *P* < 0.05) enrichment of many of the same functional pathways detected by PaintOmics (Supplemental Table 4). Additionally, categories related to polysaccharide biosynthesis (RNA-Seq), sucrose/starch metabolism (RNA-Seq), heme/virulence (RNA-Seq), nickel cation binding (RNA-Seq and proteomics), and arginine biosynthesis (secretomics) were significantly enriched in STRING analysis of these data.

**Table 2:** Pathway enrichment analysis in BRIC-23 RNA-Seq and proteomics datasets.

Pathway name^a^	Unique genes	Proteomics *P*-value	RNA-Seq Gene expression *P*-value	Combined *P*-value (Fisher)
Arginine biosynthesis	16	0.04	0.02	0.01
Purine metabolism	44	0.05	0.03	0.01
Thiamine metabolism	9	0.00	1.00	0.02
Quorum sensing	36	0.45	0.01	0.03
Phosphotransferase system (PTS)	16	0.05	0.12	0.04
Ribosome	56	0.04	1.00	0.17
Fructose and mannose metabolism	14	0.05	0.44	0.11
Two-component system	62	0.88	0.04	0.15
Riboflavin metabolism	9	0.49	0.05	0.12

^a^Significant (*P* < 0.05, Fisher exact test**)** pathway enrichments for each dataset (RNA-Seq, proteomics), as well as the combined datasets, are indicated. Pathway enrichment analysis was performed using PaintOmics 3.

### Expression changes in Agr and virulence factors in spaceflight

Notably, expression of genes comprising the *agr* operon were highly upregulated in spaceflight samples, with *agrA, agrB, agrC*, and *agrD* each showing an approximately 14-fold increase in transcript abundance, and *RNAIII* displaying an 88-fold increase in transcript abundance (Supplemental Table 1). AgrA, the direct transcriptional activator of the *agr* P2 and P3 operons, was also upregulated 3.96-fold in the FLT proteomics data (Table 1), corroborating the increased transcript abundance of *agrA* observed in the RNA-Seq data. Additionally, genes and proteins known to be upregulated by Agr were also upregulated in FLT samples in a manner consistent with a highly active Agr system, including increased expression of genes encoding PSM alpha (*psmα*, 8.08-fold) and gamma hemolysin C (*hlgC*, 2.20-fold), and increased protein abundance of SspA (6.95-fold), SspB (13.07-fold), and ScpA (7.18-fold)^27–32^. The secreted metalloprotease aureolysin (Aur), regulated by Agr via repression of Rot^30^, was also upregulated by 2.2–3.5 fold in the secretomics and proteomics datasets, respectively (Table 1). Agr has also been implicated in positive regulation of the capsular polysaccharide synthesis operon *capABCDE*^33^, and expression of these genes was upregulated in spaceflight (2.15–2.39-fold; Supplemental Table 1). Abundance of the surface protein fibronectin-binding protein A (FnbA), the expression of which is inhibited by Agr^34,35^, was also significantly decreased in the spaceflight proteomics data (−1.81-fold).

Several virulence factors not under Agr’s regulatory control were also upregulated in spaceflight samples. The Type VII-like Ess secretion system had six transcripts with increased abundance in FLT samples, four of which were also identified and upregulated in the proteomics and/or secretomics datasets. Staphylococcal nuclease (Nuc) was also highly upregulated in secretomics samples from spaceflight (7.72-fold). Although increased abundance of other non-*agr-*regulated virulence factors was also observed, such as those implicated in hemolysin production (CvfC, 1.51-fold^36^) and nasal colonization (SceD, 1.57-fold^37^), other well-characterized *S. aureus* virulence factors such as hyaluronidase, coagulase, lipase, and staphylokinase, were not identified as being significantly altered between FLT and GC samples.

### *S. aureus* metabolic alterations under spaceflight conditions

*S. aureus* is noted for its ability to thrive in a variety of environments and adjust its metabolism accordingly, and its virulence potential can be mediated by diverse environmental and nutritional stimuli^5,38^. In addition to genes and proteins related to the Agr quorum sensing system and its regulon, expression patterns of metabolic genes and proteins in spaceflight suggested that these *S. aureus* cultures may have experienced an altered metabolic state compared to the ground control cultures. Both cellular and secreted metabolites were analyzed for alterations in spaceflight cultures, and PCA plots of metabolomics data indicated that spaceflight samples and ground control samples clustered separately (Supplemental Fig. 2a). These metabolomics data also corroborated many of the changes in gene and protein expression levels associated with *S. aureus* metabolic functions. Urease expression, for example, was noted to be significantly upregulated in spaceflight, with seven genes upregulated between 4.12 and 5.02-fold and six proteins upregulated between 1.85 and 2.53-fold. Urea concentrations were decreased in spaceflight supernatant samples but did not meet the statistical cutoffs applied to the metabolomics analyses (data not shown). Additionally, altered levels of metabolites, genes and/or proteins related to arginine metabolism (increased ornithine, arginine/ornithine antiporter ArcD, Arginine deiminase ArcA, arginine kinase) were observed in FLT samples relative to GCs (Supplemental Tables 1–3, 5, 6).

Spaceflight *S. aureus* samples also exhibited several features congruent with a transition to catabolism of non-preferred carbon sources. These included decreased abundance of glucose in culture supernatants (Supplemental Table 6), decreased abundance of a glucose import protein (SAR1435), and functional enrichment (Table 1) and upregulated expression of genes and/or proteins of several sugar transporters (SAR0235, SAR0193, SAR2244, SAR1803) (Supplemental Tables 1–3). Several members of TCA cycle, including CitZ, OdhA/B, and SdhA/B displayed increased protein abundance and/or gene expression in spaceflight samples. Altered levels of metabolites, genes and/or proteins related to cysteine/serine metabolism (increased O-acetylserine, homoserine, cysteine, ornithine carbamoyltransferase, and serine dehydratase), and methionine metabolism (decreased methionine, increased methionine sulfoxide, increased methionine ABC transporter) were also observed in spaceflight samples relative to ground controls (Supplemental Tables 1–3, 5, 6). Additionally, increased expression of several metal transport genes was observed in the spaceflight cultures (Supplemental Table 1).

## Discussion

Our analysis of the *S. aureus* RNA-Seq, proteomics, secretomics, and metabolomics datasets from BRIC-23 indicate that *S. aureus* undergoes significant alterations in physiology and virulence factor production in the spaceflight environment which could alter its pathogenic potential for astronauts conducting long-term spaceflight missions. However, these results need to be interpreted in the context of the experimental confines of the BRIC-23 experiment, namely that only a single time point (48 h growth) was assessed, and that the experiment was conducted at ISS ambient temperature ( ~ 22 °C). Ground-based pre-science verification test (pre-SVT) growth curves previously conducted at ~25 °C (using dried starting inoculum on petri dishes and growth medium identical to those used in the BRIC-23 flight and ground controls) indicated that *S. aureus* entered early stationary phase by 48 h growth in this condition^20^. In comparison, SVT incubation of BRIC-PDFUs in the the ISS Environmental Simulator (ISSES) chamber at KSC conducted at ~22 °C, which more closely mimicked ISS ambient temperature, showed that cultures reached late exponential phase growth by 48 h^20^. Since a true growth curve (multiple time points) was not conducted in the actual BRIC-23 flight and ground control experiments, we cannot speculate on any potential differences in the growth rates and/or phase of growth between BRIC-23 FLT and GC cultures at the 48 h time point. However, the reported total CFUs per petri dish in both flight and ground control experiments suggest that the FLT cultures may have grown to higher cell densities compared to GC cultures (Fig. 1b). Similar to this, experiments previously conducted with *Pseudomonas aeruginosa* demonstrated that this bacterium achieved increased final cell density (as measured by flow cytometry of fixed cells) in flight cultures relative to ground controls under the specific growth medium characteristics of low phosphate and low oxygen availability^39^. *Esherichia coli* and *Bacillus subtilis* spaceflight cultures grown at 23 °C in a fluid processing apparatus also achieved increased stationary phase cell densities in flight cultures relative to their corresponding ground controls^40^. It is therefore not clear whether the *S. aureus* BRIC-23 FLT cultures were in an identical phase of growth compared to the GC cultures, which could impact some of the observed differences discussed in more detail below.

Additionally, temperature is known to have a significant impact on *S. aureus* gene expression, as it has recently been shown that expression of genes encoding secreted proteases and toxins (*aur, sspA, sspB, esxA*), as well as *agrD*, displayed increased transcript abundance at 34 °C compared to 37 °C and 40 °C^41^. Therefore, the BRIC-23 data could be considered more relevant to conditions experienced by *S. aureus* outside of the host (i.e., growth and/or persistence on fomites, which could impact *S. aureus* transmission). Future experiments examining multiple time points and a more biologically relevant incubation temperature (35–37 °C) represent logical follow-up steps to gain a complete picture of the *S. aureus* physiological response to spaceflight.

A major finding of the BRIC-23 data analysis was that the *S. aureus* Agr quorum sensing response, as well as increased expression of both Agr-dependent and Agr-independent virulence factors, was highly upregulated during this spaceflight experiment. Given the challenges of medical treatment of bacterial infections in space, increased potential for Agr quorum sensing and production of virulence factors are concerns for astronaut health, and worthy of additional study of this bacterium under spaceflight conditions. Agr function has been identified as a critical component of infection and lethality in animal models, including rabbit and mouse models of osteomyelitis, necrotic pneumonia, and skin infection^23,42,43^. Additionally, *agr* has been observed to be important for human infection, with CA-MRSA skin infections strongly affected by Agr activity^27^. However, naturally occurring *agr* mutant strains as well as mixed cultures of wildtype and mutant *agr* variants have been isolated from human infections, indicating that Agr is not the only system responsible for pathogenesis, and that loss of Agr function may in fact contribute to chronic infection^44,45^. Autoinduction of Agr is activated in late exponential phase and stationary phase in response to high cell density, as higher concentrations of secreted AIP can be recognized by AgrC, resulting in a positive feedback loop of *agr* activation. Low Agr activity is characterized by cell attachment and biofilm formation, and as cell density increases, *agr* activation encourages a cellular transition to a dispersal and pathogenesis-focused lifestyle^8,31,46–48^. This is accomplished through upregulation of secreted proteases and toxins, which help cells overcome host defenses, and downregulation of surface adhesion factors to aid in dissemination throughout the host. Given that higher CFUs were recovered from the BRIC-23 FLT cultures at 48 h growth relative to the GC cultures, it is possible that the increased cell density of FLT cultures contributed to increased activation of Agr. Interestingly, even though increased Agr activation was observed in FLT cultures, the average concentration of secreted proteins in FLT sample supernatants was approximately 30% less than the GC culture supernatants (Fig. 1c), possibly a consequence of increased expression and abundance of secreted proteases (SspA, SspB, ScpA, Aur) which are known to have significant effects on the stability of the *S. aureus* exoproteome^49–53^. Interestingly, observations from previous ground-based simulated microgravity studies of *S. aureus* also showed that simulated microgravity cultures excreted less protein compared to normal gravity controls^19^.

Several virulence factors not under Agr’s regulatory control were also upregulated in spaceflight samples. These included several components of the Type VII-like Ess secretion system, which has been implicated in protection from host antimicrobial resistance^54^ as well as exhibiting a potential role in cross-strain competition^24^. Therefore, increased expression of the Ess system could potentially confer a competitive advantage to *S. aureus* in colonizing and/or persisting on environmental fomites, as well as the human body. Staphylococcal nuclease, controlled by the SaeRS regulatory system^55,56^, was also highly upregulated in secretomics samples from spaceflight. This enzyme breaks down extracellular DNA and RNA^57^, contributing to biofilm dispersal^58^. Nuclease also contributes to *S. aureus* pathogenicity by enhancing its ability to evade killing by neutrophils, via degradation of the DNA backbone of neutrophil extracellular traps (NETs)^59^. Increased abundance of other non-*agr-*regulated virulence factors implicated in hemolysin production (CvfC^36^) and nasal colonization (SceD^37^) was also observed. Collectively, this data suggests that the virulence potential of *S. aureus* in spaceflight could be amplified by factors in addition to the Agr regulon.

Additionally, *S. aureus* appears to undergo an altered metabolism during spaceflight. For example, urease subunit gene expression and/or protein abundance was significantly upregulated in spaceflight. This enzyme hydrolyzes urea to form ammonia and carbon dioxide, and is a vital contributor to acid stress survival in *S. aureus*^60^. Increased urease expression and activity, in conjunction with increased acid production, has also been demonstrated in *S. aureus* biofilms^61^. Altered levels of metabolites, genes and/or proteins related to arginine metabolism were also observed in spaceflight samples relative to ground controls. Collectively, these changes could reflect an overall increase in urea cycle activity in response to acid stress and/or as an alternative pathway for ATP production, as the arginine deiminase pathway produces both ammonia and ATP (via substrate-level phosphorylation)^62,63^. Spaceflight *S. aureus* samples also exhibited several features congruent with a transition to catabolism of non-preferred carbon sources, including decreased abundance of extracellular glucose, increased abundance of gene transcripts and/or proteins encoding various sugar transporters, and increased protein abundance and/or gene expression of TCA cycle enzymes. Derepression of the tricarboxylic acid (TCA) cycle in *S. aureus* has been associated with decreased availability of carbon sources which generally occurs during post-exponential growth^64,65^. Additionally, altered levels of metabolites, genes and/or proteins related to cysteine/serine metabolism (increased O-acetylserine, homoserine, cysteine, ornithine carbamoyltransferase, and serine dehydratase), and methionine metabolism (decreased methionine, increased methionine sulfoxide, increased methionine ABC transporter) were observed in spaceflight samples relative to ground controls (Supplemental Tables 1–3, 5, 6). These changes, in combination with increased expression of several metal transport genes (Supplemental Table 1), suggest that *S. aureus* could have altered sulfur and/or metal requirements to support its metabolism under flight conditions.

The global regulator CodY was also identified in the cellular proteomics analysis, with a –1.52-fold change in spaceflight samples. CodY notably responds to changes in nutrient levels and alters the expression of a wide range of metabolic genes, virulence factors, and other regulators^56,66–68^. Some targets known to be repressed by this regulator include capsule synthesis, Agr, and staphylococcal nuclease, which all exhibit increased abundance in spaceflight samples (Supplemental Tables 1–3). Therefore, depleted nutrients in the spaceflight environment could contribute to these observed alterations in *S. aureus* virulence factor production and metabolism, partly through decreased CodY activity.

**Fig. 5:**
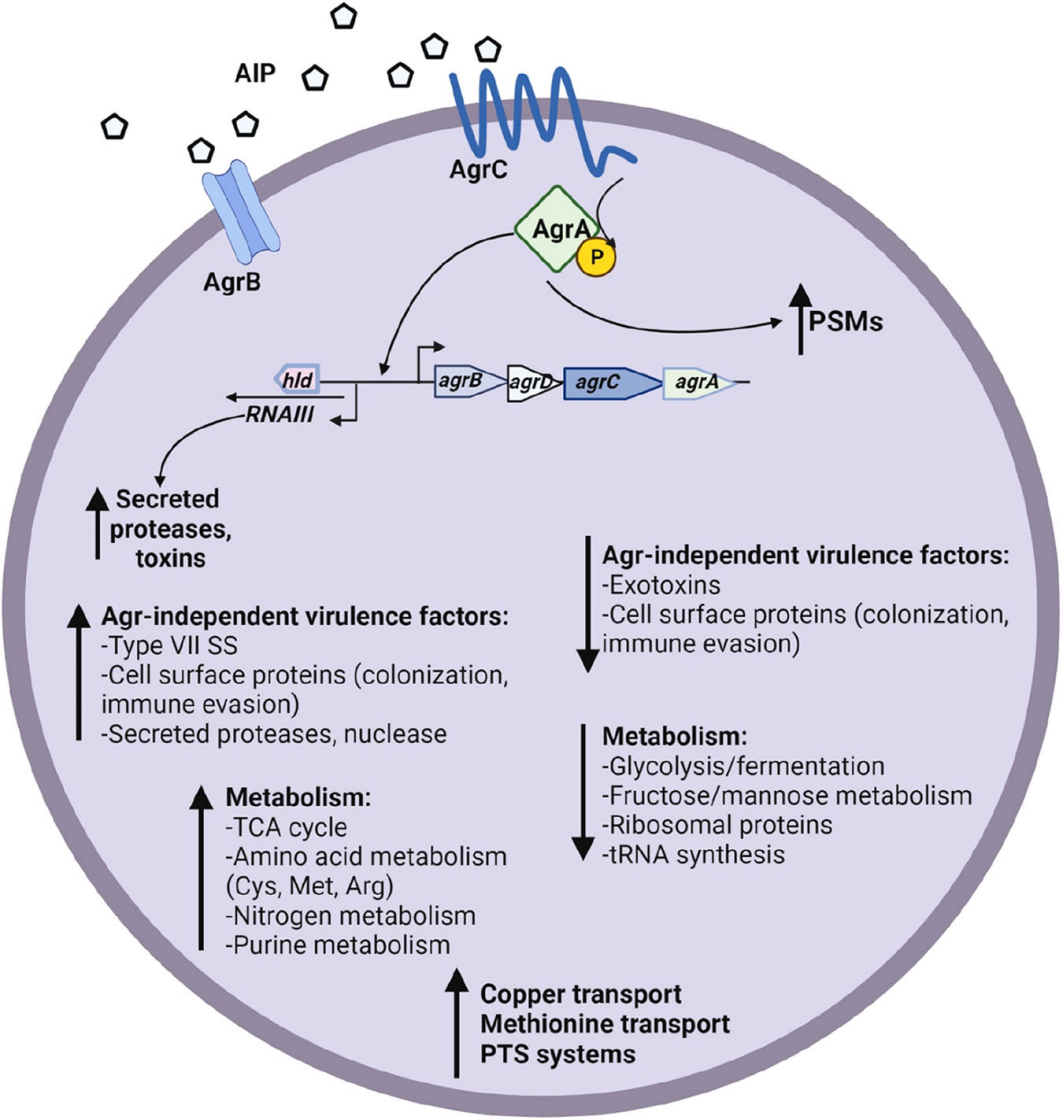
Summary of *S. aureus* alterations in physiology and virulence factor production in the spaceflight environment. Diagram is based on analyzed RNA-Seq, proteomics, secretomics, and metabolomics data presented in this study.

In summary, data from the BRIC-23 experiment indicate that *S. aureus* spaceflight cultures exhibit changes in physiology and virulence factor production (summarized in Fig. 5) which could affect its ability to cause human disease during long-duration spaceflight missions. Specifically, *S. aureus* spaceflight samples may have altered metabolism (increased amino acid metabolism, TCA cycle, and transport of non-preferred carbon sources, and decreased glycolysis/fermentation and translation machinery) relative to ground-control cultures. Furthermore, both Agr-dependent and Agr-independent virulence factor expression were upregulated in spaceflight, with the *agr RNAII* and *RNAIII* transcripts themselves being the most highly upregulated. However, it is important to interpret these results in the context of the experimental confines of the BRIC-23 experiment.

## Methods

### Overview of BRIC-23 experimental design

A general overview of the BRIC-23 flight (FLT) experiment is outlined in Fig. 1, and complete details have been previously described^20,21^. In brief, two BRIC-PDFUs (each containing five 60 mm Petri dishes with 10^7^ CFU of *S. aureus* UAMS-1^23^ cells dried on the bottom of each plate) were launched to the ISS on SpaceX CRS-9 on 07/18/2016. Growth was actuated in each petri dish by addition of 8.5 ml tryptone soytone yeast extract medium containing 10% glycerol (TSYG) on 07/22/2016, followed by growth at ~22 °C for 48 h. BRIC-PDFUs were then placed in −80 °C stowage until return from the ISS on 08/30/2016. Samples remained frozen during return and were stored at −80 °C until deintegration. Ground control (GC) experiments were performed asynchronously with the same hardware, configuration, timing, and growth temperature as in the BRIC-23 FL experiment. Deintegrated samples from both FLT and GC were stored at −80 °C until processed for RNA-Seq, proteomics, and metabolomics as described in^21^. RNA-Seq, cellular proteomics, and metabolomics raw data files and associated metadata are available through NASA Open Science for Life in Space (OSDR) entry OSD-145: BRIC-23 GeneLab Process Verification Test: *Staphylococcus aureus* transcriptomic, proteomic, and metabolomic data (10.26030/ga0p-2817) (https://osdr.nasa.gov/bio/repo/data/studies/OSD-145).

### BRIC-23 secretomics and data analysis

Filter-sterilized and concentrated (5 kDa MW cutoff) BRIC-23 supernatants were provided by NASA’s Life Science Data Archive (LSDA) (*n* = 5 each of BRIC-23 FLT and GC samples). Proteins from a 480–610 µl volume of each sample were precipitated by adding 120–150 µL of 100% (v/v) trichloroacetic acid to each sample, followed by incubation for 30 min at 4 °C. Samples were centrifuged at 20,000 × *g* for 5 min, then the supernatant was discarded. Protein pellets were washed with 200 µL of cold acetone, then samples were centrifuged again as above, and the acetone wash was repeated for a total of two washes. Protein pellets were dried by leaving tubes open in a Class 2AII biosafety cabinet for 5 min to allow acetone to evaporate. Next, pellets were resuspended in 200 µL urea buffer (6 M urea, 0.4 M Tris, pH 7.8) and stored at −20 °C. Protein concentrations were measured via Bio-Rad Protein Assay (Bio-Rad Protein Assay Kit, Bio-Rad).

Equal amounts of protein (10.59 µg per sample) were used, and three replicates per sample type were separated by sodium dodecyl sulfate-polyacrylamide gel electrophoresis (SDS-PAGE). The entire lane per sample was excised with a scalpel and diced into 1 mm^2^ cubes. In-gel trypsin digestion was performed as previously described^69,70^. Protein identification and quantification were performed using a label-free quantitative shotgun mass proteomics approach using an HPLC-Orbitrap Fusion mass spectrometer (UF-ICBR proteomics core). Briefly, the peptide samples were analyzed using a 250-mm Ultrahigh-Performance Liquid Chromatography (UHPLC) system coupled to an Orbitrap Fusion mass spectrometer (Thermo Scientific). The Thermo EASY nano-LC system was used for liquid chromatography, employing a 20-mm C16 pre-column (Thermo Scientific) to remove impurities. A reversed-phase C18 analytical column with a 100 Å pore (Thermo Scientific, Acclaim PepMap 100 C18 LC Column) was used for sample separation. The following solvents were used for chromatography: solvent A (0.1% formic acid), solvent B (80% acetonitrile, 0.1% formic acid) with a 2–40% solvent B acetonitrile gradient for 105 min, followed by a 14-minute wash with 98% solvent B, and equilibration with 2% solvent A. The LC system was directly interfaced with the Orbitrap Fusion mass spectrometer. The Orbitrap detector acquired MS data at 120 K resolution with a scan range of 350–2000 m/z. For MS/MS analysis, ions were isolated by a quadrupole, prioritizing the most intense ions, and ions for all available parallelizable times were injected. Precursor ions were then excluded for 36 s. Fragmentation was performed using collision-induced dissociation (CID) at a collision energy of 35% and an activation time of 10 ms.

Proteins were identified and quantified from the generated raw data using Proteome Discoverer, as previously described^69,71–78^. These data were also analyzed by Scaffold software version 4.11.0 (Proteome Software, Inc., USA) to identify secreted proteins with statistically significant alterations in expression. Briefly, tandem mass spectra were extracted, charge state deconvoluted, and deisotoped using Proteome Discoverer (Thermo Fisher Scientific). Tandem mass spectrometry (MS/MS) samples were analyzed by using the SEQUEST algorithm (Thermo Fisher) using available databases containing *S. aureus* proteins (Genbank #BX571856.1) and contaminants. Scaffold software version 4.11.0 (Proteome Software, Inc., USA) was used to validate MS/MS-based peptide and protein identifications, where the required delta Cn scores were >0.2 and XCorr scores were >1.2, 1.9, 2.3, and 2.6 for singly, doubly, triply, and quadruply charged peptides, respectively. Protein identifications were accepted if they were established at >95.0% probability and contained >2 identified peptides, with a peptide FDR of 0.2%. The protein probabilities were assigned by the Protein Prophet algorithm. Weighted spectral counts were used for protein quantification, and data were normalized before the fold changes were calculated between the flight and control samples. The P < 0.05 (Student t-test, calculated in GraphPad Prism) and a minimum 1.5-fold change between treatment and control proteins indicated proteins with significant changes in abundance. Secretomics raw data files and associated metadata are available through NASA OSDR entry OSD-500: BRIC-23: Secretomics (10.26030/rztr-e997) (https://osdr.nasa.gov/bio/repo/data/studies/OSD-500).

### BRIC-23 cellular proteomics analysis

Cellular proteins were identified and quantified from the generated raw data from the BRIC-23 experiment (https://osdr.nasa.gov/bio/repo/data/studies/OSD-145) using Proteome Discoverer. Briefly, tandem mass spectra were extracted, charge state deconvoluted, and deisotoped using Proteome Discoverer (Thermo Fisher Scientific). Tandem mass spectrometry (MS/MS) samples were analyzed by using the SEQUEST algorithm (Thermo Fisher) using available databases containing *S. aureus* proteins (Genbank #BX571856.1). All analyzed fractions were merged before the analysis. SEQUEST search parameters were as follows: two maximum trypsin mis-cleavages, precursor mass tolerance of 10 ppm, fragment mass tolerance of 0.6 Da; static modifications were TMT six-plex/+229.163 Da (N-terminus, Lys) and carbamidomethyl modification/+57.021 Da (Cys); dynamic modification was oxidation modification/+15.995 Da (Met). Maximum dynamic modifications per peptide were four. High XCorr Confidence Thresholds were 1.2, 1.9, 2.3, and 2.6 for z = 1, 2, 3, and >4, respectively. The maximum allowable delta Cn value was 0.05. Moreover, a decoy databank search was performed to establish FDR at a minimum of 0.05 for protein identifications, where the validation was done using the q-value method. All the medium and high-confidence peptides were used to identify and quantify proteins. The reporter ions (i.e., m/z 126, 127 N, 127 C, 128 N, 131) were identified where the most confident centroid was used and 10 ppm for reporter ion mass tolerance. The reporter ion values were normalized to control samples (128 N). Proteins belonging to multiple protein groups were grouped into a single accession number, and final ratios were reported. Fold changes were calculated between flight and ground samples, where the P-value was calculated using the Student’s t-test (*P* < 0.05), indicating proteins with significant changes in abundance (minimum 1.5-fold change).

### BRIC-23 RNA-Seq analysis

FastQ data files from *n* = 9 FLT and *n* = 9 GC samples were downloaded from OSDR (https://osdr.nasa.gov/bio/repo/data/studies/OSD-145) and imported into CLC Genomics Workbench (Qiagen) for analysis. Ribosomal RNA reads were filtered out, and the remaining reads were mapped to the *S. aureus* MRSA252 genome (Genbank #BX571856.1). The UAMS-1 genome (strain used in the BRIC-23 flight experiment) is not closed. Therefore, the updated Genbank genome file for MRSA252, which contains annotations for sRNAs^79^ and critical virulence genes such as phenol soluble modulins (*psm*_*a1-4*_), was used instead. A previously published analysis pipeline was followed^79,80^ using the “RNA-Seq analysis” feature of CLC Genomics Workbench version 21, with quantile normalization of data sets^81^. Standard cutoffs ( ≥ 2-fold change, mean normalized expression value ≥ 10 for both samples) were used to curate differential gene expression (DE) data, performed as described in^82^. Microsoft Excel was used for RNA-Seq DE data reduction and statistical analysis. Student’s two-tailed t-test was used to determine significance (*P* < 0.000016 with 5% FDR Bonferroni correction).

### Metabolomics Analysis

MetaboAnalyst 5.0^83^ (https://www.metaboanalyst.ca/home.xhtml) was used to quantify the cellular and supernatant untargeted metabolomics data from BRIC-23 datasets (https://osdr.nasa.gov/bio/repo/data/studies/OSD-145). All missing values were replaced by half of the minimum positive value found within the data. However, features with over 50% missing values were removed from the dataset. The data were normalized by sum and subjected to log transformation and auto-scaling, where the values were mean-centered and divided by the standard deviation of each variable. A P-value threshold of 0.05 (Student t-test) was used for generating volcano plots and a fold change of 1.5.

### Bioinformatics

Functional categories of genes/proteins identified by RNA-Seq, proteomics, and secretomics as being statistically significant ( > 2-fold change in expression for RNA-Seq, and >1.5-fold change for proteomics and secretomics) were assigned using manual curation based on gene annotation and/or predicted function using the following databases: Aureowiki^84^, Uniprot^85^, PATRIC^86^ and Biocyc^87^. Hierarchical clustering and heat map generation of statistically significant DE data was performed using 1-Pearson correlation on rows and columns, using Morpheus default settings (https://software.broadinstitute.org/morpheus). Venn diagram of overlapping and unique DE genes/proteins identified by RNA-Seq, proteomics, and secretomics as being statistically significant with > 2-fold change in expression for RNA-Seq and > 1.5-fold change for proteomics and secretomics, was generated using OmicsBox (BioBam, Valencia, Spain)^88^. Principal Components Analysis (PCA) of RNA-Seq, proteomics, and secretomics data was performed using ClustVis^89^. PCA plots for metabolomics data were generated with Rscript chemometrics.R within the MetaboAnalyst program^83^. RNA-Seq, cellular proteomics, and/or secretomics DE data were analyzed using Paint-omics version 3^25^ and STRING version 11.5^26^ to detect enriched KEGG pathways and genes/proteins, respectively. The NCBI MRSA252 genome (Genbank #BX571856.1) was used as a reference in both analyses.

### Statistical analysis

Statistical analysis specific to each -omics analysis is described in each respective section above. Statistical analyses were performed using Microsoft Excel, Graphpad Prism 9, Scaffold software version 4.11.0, or MetaboAnalyst 5.0, as indicated.

### Reporting summary

Further information on research design is available in the Nature Research Reporting Summary linked to this article.

### Supplementary information

Supplemental Material

Reporting Summary

## Data Availability

All BRIC-23 -omics datasets and corresponding metadata can be accessed through NASA OSDR (for RNASeq, proteomics, and metabolomics: 10.26030/ga0p-2817f; or secretomics: 10.26030/rztr-e997). All other data presented in this manuscript is available as figures/tables, or supplementary figures/tables.
