# Supplementary material for: Altered quorum sensing and physiology of Staphylococcus aureus during spaceflight detected by multi-omics data analysis
Source: NPJ Microgravity. 2024 Jan 8;10:2. doi: 10.1038/s41526-023-00343-7 (PMC10774393; doi:10.1038/s41526-023-00343-7)
Supplement: Supplementary file 1 — Supplemental Material [file 41526_2023_343_MOESM1_ESM.pdf]

Altered Quorum Sensing and Physiology of *Staphylococcus aureus* During Spaceflight Detected by Multi-omics Data Analysis

Matthew R. Hauserman[1], Mariola J. Ferraro[1], Ronan K. Carroll[2], and Kelly C. Rice[1*]

[1]Department of Microbiology and Cell Science, IFAS, University of Florida, Gainesville, Florida, USA

[2]Department of Biological Sciences, Ohio University, Athens, OH, USA

*Corresponding author: Department of Microbiology and Cell Science, IFAS, University of Florida, Gainesville, Florida, USA, kcrice@ufl.edu.

Supplemental Data File

(Contains Supplemental Tables 1-6, and Supplemental Figures 1-2)

Supplemental Table 1. RNA-Seq data from spaceflight (FLT) vs. ground control (GC) cultures

PROTEIN ID	GENE ID	FUNCTION	P VALUE	RATIO (FL/GC)
	SARs022	RNAIII; effector small RNA for agr system	1.43E-07	87.90
YP_041487.1	SAR2124	Accessory gene regulator D (pheromone precursor, type III)	5.11E-06	14.76
YP_041488.1	SAR2125	Accessory gene regulator C (sensor histidine kinase)	3.44E-09	14.43
YP_041489.1	SAR2126	Accessory gene regulator A (response regulator)	4.65E-10	14.30
YP_041486.1	SAR2123	Accessory gene regulator B	2.82E-06	13.90
	psma	phenol-soluble modulin alpha	1.33E-05	8.08
YP_040561.1	SAR1150	hypothetical protein	3.50E-06	5.80
YP_041728.1	SAR2373	Urease beta subunit (EC 3.5.1.5)	1.42E-06	5.02
YP_041729.1	SAR2374	Urease alpha subunit (EC 3.5.1.5)	6.94E-06	4.91
YP_039750.1	SAR0286	hypothetical protein	1.87E-06	4.82
YP_039748.1	SAR0284	Type VII secretion system protein EssC	1.04E-05	4.73
YP_041730.1	SAR2375	Urease accessory protein UreE	1.82E-06	4.66
YP_041727.1	SAR2372	Urease gamma subunit (EC 3.5.1.5)	1.56E-07	4.65
YP_039743.1	SAR0279	6 kDa early secretory antigenic target ESAT-6 (EsxA)	1.01E-05	4.61
YP_041966.1	SAR2621	Holin-like protein CidA	1.59E-06	4.44
YP_041732.1	SAR2377	Urease accessory protein UreG	9.88E-06	4.37
YP_041731.1	SAR2376	Urease accessory protein UreF	5.97E-06	4.37
YP_039747.1	SAR0283	Type VII secretion protein EssB	8.56E-06	4.28
YP_039603.1	SAR0138	Purine nucleoside phosphorylase (EC 2.4.2.1)	2.23E-10	4.24
YP_041733.1	SAR2378	Urease accessory protein UreD	8.83E-07	4.12
YP_039601.1	SAR0136	Predicted cell-wall-anchored protein SasD (LPXAG motif)	8.54E-06	3.77
YP_039657.1	SAR0192	N-acetylmuramic acid 6-phosphate etherase (EC 4.2.1.126)	9.31E-06	3.76
YP_039731.1	SAR0268	Putative ribose uptake protein RbsU, GRP transporter family (TC 2.A.7.5)	8.76E-06	3.69
YP_040040.1	SAR0592	hypothetical protein	8.51E-06	3.43
YP_039730.1	SAR0267	D-ribose pyranase (EC 5.4.99.62)	3.69E-08	3.31
YP_041592.1	SAR2232	oxidoreductase ylbE	2.74E-06	3.29
YP_039746.1	SAR0282	Putative secretion accessory protein EsaB/YukD	9.16E-06	3.24
YP_041602.1	SAR2244	PTS system, mannitol-specific IIC component / PTS system, mannitol-specific IIB component (EC 2.7.1.197)	1.78E-06	3.21
YP_039636.1	SAR0171	hypothetical protein	1.53E-06	3.04
YP_039744.1	SAR0280	Type VII secretion protein EsaA	1.41E-08	3.01
YP_039742.1	SAR0278	Secreted antigen precursor; staphyloxanthin biosynthesis	7.08E-06	3.00
YP_039656.1	SAR0191	Outer surface protein of unknown function, cellobiose operon	6.87E-06	2.98
YP_039729.1	SAR0266	Ribokinase (EC 2.7.1.15)	5.67E-06	2.93
YP_041982.1	SAR2637	Copper-exporting P-type ATPase A CopA	9.93E-07	2.91
YP_039745.1	SAR0281	Putative secretion system component EssA	4.32E-06	2.91
YP_040007.1	SAR0558	L-threonine 3-dehydrogenase (EC 1.1.1.103)	3.29E-06	2.81
YP_041956.1	SAR2611	L-serine dehydratase, beta subunit (EC 4.3.1.17)	1.78E-07	2.76
YP_039604.1	SAR0139	Tetracycline resistance, MFS efflux pump => Tet(38)	2.54E-06	2.69
YP_041594.1	SAR2234	Zn(II) and Co(II) transmembrane diffusion facilitator	1.19E-07	2.68
YP_041593.1	SAR2233	Zn(II) or Co(II)-specific transcriptional repressor protein	1.20E-05	2.63
YP_042010.1	SAR2668	Ribulosamine/erythrulosamine 3-kinase potentially involved in protein deglycation	2.60E-07	2.61
	SAR2506	Phosphoglycerate mutase	1.00E-07	2.55
YP_041951.1	SAR2605	D-lactate dehydrogenase (EC 1.1.1.28)	1.55E-05	2.54
YP_041767.1	SAR2413	Oxidoreductase, short-chain dehydrogenase/reductase family	1.10E-08	2.53
YP_039700.1	SAR0235	PTS system, N-acetylmannosamine-specific IIC component / PTS system, N-acetylmannosamine-specific IIB component	1.73E-06	2.51
YP_039484.1	SAR0007	ADP-dependent (S)-NAD(P)H-hydrate dehydratase (EC 4.2.1.136)	5.89E-08	2.46
YP_039635.1	SAR0170	Cobalt-zinc-cadmium resistance protein	7.35E-07	2.41
YP_041957.1	SAR2612	Transcriptional regulator pfoR	1.48E-07	2.41
YP_039616.1	SAR0151	Capsular polysaccharide synthesis enzyme Cap5A / Tyrosine-protein kinase transmembrane modulator EpsC	1.52E-05	2.39
YP_039658.1	SAR0193	PTS system, N-acetylmuramic acid-specific IIB component (EC 2.7.1.192) / PTS system, N-acetylmuramic acid-specific IIC component	8.43E-07	2.39
YP_039617.1	SAR0152	Tyrosine-protein kinase EpsD (EC 2.7.10.2) / Capsular polysaccharide synthesis enzyme Cap5B	3.30E-06	2.30
	SARs049	related to mntABC operon for manganese import	1.14E-07	2.29
YP_040492.1	SAR1079	Manganese import protein MntH	1.69E-06	2.26
YP_041277.1	SAR1895	hypothetical protein	6.65E-08	2.21
YP_041860.1	SAR2510	Cytolytic pore-forming protein S component => Gamma-hemolysin HlgC	2.04E-07	2.20
YP_041955.1	SAR2610	L-serine dehydratase, alpha subunit (EC 4.3.1.17)	6.51E-08	2.19
YP_040148.1	SAR0720	Lead, cadmium, zinc and mercury transporting ATPase (EC 3.6.3.3) (EC 3.6.3.5); Copper-translocating P-type ATPase (EC 3.6.3.4)	7.34E-08	2.19
YP_039760.1	SAR0301	hypothetical protein	6.45E-07	2.18
YP_039618.1	SAR0153	Capsular polysaccharide synthesis enzyme Cap8C; Manganese-dependent protein-tyrosine phosphatase (EC 3.1.3.48)	8.70E-08	2.17
YP_042117.1	SAR2780	hypothetical protein	8.43E-08	2.17
YP_039619.1	SAR0154	Probable polysaccharide biosynthesis protein EpsC	3.85E-07	2.16

YP_041726.1	SAR2371	Eukaryotic-type low-affinity urea transporter	4.03E-06	2.15
YP_039620.1	SAR0155	capsular polysaccharide synthesis enzyme	2.52E-11	2.15
YP_041358.1	SAR1984	Bacterial non-heme ferritin (EC 1.16.3.2)	2.41E-07	2.13
YP_041769.1	SAR2417	Urocanate hydratase (EC 4.2.1.49)	4.63E-07	2.11
YP_039659.1	SAR0194	Transcriptional regulator, RpiR family	1.64E-08	2.09
YP_041889.1	SAR2538	Choline ABC transport system, ATP-binding protein OpuBA	1.42E-06	2.08
YP_042112.1	SAR2775	2-oxoglutarate/malate translocator-like protein	1.92E-07	2.05
YP_041628.1	SAR2275	Hypothetical protein SAV2184	3.02E-08	2.04
YP_042067.1	SAR2725	Predicted cell-wall-anchored protein SasF (LPXAG motif)	6.25E-12	2.03
YP_041440.1	SAR2070	Hypothetical protein, SAV0881 homolog [SA bacteriophages 11, Mu50B]	5.43E-06	2.03
YP_040827.1	SAR1425	2-oxoglutarate dehydrogenase E1 component (EC 1.2.4.2)	1.59E-12	2.03
YP_041237.1	SAR1851	Riboflavin biosynthesis protein RibBA; GTP cyclohydrolase II	1.66E-11	2.02
YP_041238.1	SAR1852	Riboflavin synthase eubacterial/eukaryotic (EC 2.5.1.9)	2.25E-11	2.01
YP_040557.1	SAR1144	Arginine/ornithine antiporter ArcD	4.16E-11	2.00
YP_041780.1	SAR2428	hypothetical protein	9.41E-09	2.00
YP_041366.1	SAR1993	Aspartyl-tRNA(Asn) (EC 6.3.5.6)/Glutamyl-tRNA(Gln) amidotransferase subunit C (EC 6.3.5.7)	3.27E-09	-2.05
YP_040347.1	SAR0925	hypothetical protein	7.80E-06	-2.06
YP_040753.1	SAR1347	GMP reductase (EC 1.7.1.7)	6.28E-09	-2.07
YP_040297.1	SAR0875	FIG028593: uncharacterized membrane protein	1.02E-06	-2.11
YP_040856.1	SAR1455	hypothetical protein	1.40E-06	-2.12
	SARs292	small RNA	1.09E-05	-2.15
YP_041349.1	SAR1974	Cell envelope stress response system LiaFSR, response regulator LiaR(VraR)	2.46E-08	-2.18
	SARs228	small RNA	3.15E-10	-2.20
YP_041351.1	SAR1976	Membrane protein LiaF(VraT), specific inhibitor of LiaRS(VraRS) signaling pathway	1.09E-12	-2.22
	SARs265	small RNA	2.25E-08	-2.22
YP_042111.1	SAR2774	Collagen binding protein Cna	2.59E-09	-2.22
YP_041294.1	SAR1920	Exotoxin, phage associated	3.75E-07	-2.25
YP_041350.1	SAR1975	Cell envelope stress response system LiaFSR, sensor histidine kinase LiaS(VraS)	7.42E-09	-2.26
YP_039833.1	SAR0376	Hypothetical SAV0792 homolog in superantigen-encoding pathogenicity islands SaPI	4.23E-07	-2.27
YP_041361.1	SAR1987	Hypothetical protein SAV1896	2.25E-08	-2.27
	SAR1128	formyl peptide receptor-like 1 inhibitory protein (frame-shifted in MRSA252)	2.81E-06	-2.27
YP_039791.1	SAR0332	hypothetical protein	1.15E-05	-2.28
YP_041990.1	SAR2648	Secretory antigen precursor SsaA	2.91E-09	-2.29
YP_041620.1	SAR2267	Heme ABC type transporter HtsABC, permease protein HtsB	4.02E-08	-2.30
YP_040541.1	SAR1127	Hypothetical protein, similarity with fibrinogen-binding protein Efb	4.11E-06	-2.36
YP_040028.1	SAR0580	Long-chain-fatty-acid--CoA ligase (EC 6.2.1.3)	9.82E-06	-2.38
YP_041795.1	SAR2442	Membrane protein TcaA associated with Teicoplanin resistance	2.63E-07	-2.46
YP_039834.1	SAR0377	Hypothetical SAV0794 homolog in superantigen-encoding pathogenicity islands SaPI	2.26E-07	-2.52
YP_041295.1	SAR1921	Exotoxin, phage associated	6.37E-07	-2.55
YP_041621.1	SAR2268	Heme ABC type transporter HtsABC, heme-binding protein	2.93E-09	-2.58
YP_040586.1	SAR1175	Uracil permease @ Uracil:proton symporter UraA	6.54E-06	-2.62
YP_040587.1	SAR1176	Aspartate carbamoyltransferase (EC 2.1.3.2)	6.14E-06	-2.78
YP_041293.1	SAR1919	Exotoxin, phage associated @ Superantigen enterotoxin SEI	4.89E-08	-2.84
	SARs156	small RNA	2.62E-12	-3.01
	SARs086	small RNA	9.25E-09	-4.52
YP_039900.1	SAR0452	NADH dehydrogenase, subunit 5	3.30E-13	-8.28
YP_039901.1	SAR0453	Hypothetical transmembrane protein coupled to NADH-ubiquinone oxidoreductase chain 5 homolog	5.23E-12	-12.77

Supplemental Table 2. Cellular proteomics data from spaceflight (FLT) vs. ground control (GC) cultures

PROTEIN ID	GENE ID	FUNCTION	P VALUE	RATIO (FL/GC)
CAG41381.1	SAR2399	putative transcription regulator; phosphosugar-binding transcriptional regulator, RpiR family	0.0020	5.35
CAG39877.1	SAR0870	Methionine ABC transporter ATP-binding protein	0.0098	4.00
CAG41107.1	SAR2126	Accessory gene regulator A (response regulator)	0.0005	3.96
CAG40026.1	SAR1022	Zinc metalloproteinase aureolysin; Aur	0.0253	3.48
CAG41694.1	SAR2716		0.0409	3.27
CAG41617.1	SAR2639	CopZ putative heavy-metal-associated protein	0.0002	3.11
CAG39305.1	SAR0278	Secreted antigen precursor; staphyloxanthin biosynthesis	0.0001	2.71
CAG39731.1	SAR0720	Lead, cadmium, zinc and mercury transporting ATPase (EC 3.6.3.3) (EC 3.6.3.5); Copper-translocating P-type ATPase (EC 3.6.3.4)	0.0001	2.55
CAG39310.1	SAR0283	Type VII secretion protein EssB	0.0103	2.55
CAG40025.1	SAR1021	Cysteine protease precursor; SspB	0.0252	2.55
CAG41616.1	SAR2637	Copper-exporting P-type ATPase A CopA	0.0026	2.55
CAG41359.1	SAR2378	Urease accessory protein UreD	0.0046	2.53
CAG39314.1	SAR0287	hypothetical protein SAR0287	0.0002	2.52
CAG41651.1	SAR2674	CocE/NonD family hydrolase	0.0117	2.39
CAG39049.1	SAR0021	putative exported protein	0.0064	2.36
CAG40128.1	SAR1125	metallophosphoesterase	0.0152	2.30
CAG41355.1	SAR2374	Urease alpha subunit (EC 3.5.1.5)	0.0019	2.28
CAG41357.1	SAR2376	Urease accessory protein UreF	0.0053	2.27
CAG39284.1	SAR0258	DNA-binding response regulator LytR	0.0014	2.26
CAG41693.1	SAR2715	arginine repressor family protein	0.0287	2.21
CAG40937.1	SAR1950	putative membrane protein	0.0167	2.19
CAG39430.1	SAR0406	xanthine phosphoribosyltransferase	0.0000	2.18
CAG39879.1	SAR0872	MetQ/NlpA family ABC transporter substrate-binding protein	0.0001	2.17
CAG41354.1	SAR2373	Urease beta subunit (EC 3.5.1.5)	0.0032	2.15
CAG40051.1	SAR1048	Phosphoribosylamine--glycine ligase; Phosphoribosylglycinamide synthetase	0.0047	2.11
CAG41590.1	SAR2611	L-serine dehydratase, beta subunit (EC 4.3.1.17)	0.0247	2.10
CAG39454.1	SAR0433	type I restriction-modification system subunit M	0.0017	2.10
CAG41692.1	SAR2714	Arginine deiminase	0.0472	2.05
CAG40415.1	SAR1418	conserved hypothetical protein	0.0018	2.03
CAG39307.1	SAR0280	Type VII secretion protein EsaA	0.0059	2.03
CAG39421.1	SAR0398	Alkyl hydroperoxide reductase subunit F	0.0003	2.00
CAG39288.1	SAR0262	GntR family transcriptional regulator	0.0069	1.95
CAG41750.1	SAR2775	2-oxoglutarate/malate translocator-like protein	0.0096	1.94
CAG39732.1	SAR0721	Multicopper oxidase protein	0.0034	1.93
CAG41356.1	SAR2375	Urease accessory protein UreE	0.0020	1.91
CAG40394.1	SAR1397	putative peptidase	0.0000	1.90
CAG39890.1	SAR0884	DUF72 domain-containing protein	0.0235	1.87
CAG39050.1	SAR0022	metallo-beta-lactamase superfamily protein	0.0071	1.87
CAG39433.1	SAR0409	GMP synthase [glutamine-hydrolyzing]; GMP synthetase	0.0000	1.86
CAG41358.1	SAR2377	Urease accessory protein UreG	0.0019	1.85
CAG40125.1	SAR1122	putative succinate dehydrogenase iron-sulfur protein	0.0000	1.83
CAG41699.1	SAR2721	mannose-6-phosphate isomerase, class I	0.0116	1.79
CAG40735.1	SAR1744	DNA-3-methyladenine glycosylase I	0.0001	1.77
CAG39134.1	SAR0108	amidohydrolase; putative peptidase	0.0034	1.76
CAG41596.1	SAR2617	acyl-CoA thioesterase	0.0012	1.74
CAG41370.1	SAR2389	putative D-isomer specific 2-hydroxyacid dehydrogenase	0.0000	1.74
CAG41007.1	SAR2022	putative membrane protein	0.0060	1.74
CAG39730.1	SAR1828	transposon resolvase	0.0040	1.73
CAG40842.1	SAR1851	Riboflavin biosynthesis protein RibBA; GTP cyclohydrolase II	0.0315	1.73
CAG39567.1	SAR0546	class I SAM-dependent methyltransferase	0.0040	1.72
CAG40569.1	SAR1573	site-specific tyrosine recombinase XerD	0.0068	1.72
CAG39432.1	SAR0408	Inosine-5'-monophosphate dehydrogenase;	<0.000001	1.72
CAG39311.1	SAR0284	Type VII secretion system protein EssC	0.0005	1.71
CAG41333.1	SAR2352	GTP 3',8-cyclase; Molybdenum cofactor biosynthesis protein A	0.0276	1.69
CAG41514.1	SAR2533	putative 2-dehydropantoate 2-reductase	0.0002	1.69
CAG40479.1	SAR1481	hypothetical protein SAR1481	0.0109	1.69
CAG41360.1	SAR2379	staphylococcal accessory regulator A homologue	0.0044	1.67
CAG41407.1	SAR2427	ABC transporter ATP-binding protein	0.0040	1.66
CAG39688.1	SAR0671	putative ABC transporter protein	0.0001	1.66
CAG39216.1	SAR0189	putative thiamine pyrophosphate enzyme	0.0008	1.66
CAG39498.1	SAR0476	acetyltransferase (GNAT) family protein	0.0088	1.65
CAG39775.1	SAR0765	7-cyano-7-deazaguanine synthase; Queuosine biosynthesis protein QueC	0.0275	1.64
CAG41611.1	SAR2632	fatty acid efflux MMPL transporter FarE	0.0146	1.64
CAG40927.1	SAR1940	putative histidine kinase	0.0066	1.64
CAG41004.1	SAR2019	putative membrane protein	0.0163	1.64
CAG41589.1	SAR2610	L-serine dehydratase, alpha subunit (EC 4.3.1.17)	0.0270	1.63
CAG40861.1	SAR1870	Methionine adenosyltransferase	0.0012	1.63
CAG39519.1	SAR0497	putative pur operon repressor	0.0000	1.63
CAG39590.1	SAR0569	putative glycosyl transferase	0.0013	1.63

CAG39255.1	SAR0228	putative glutamine amidotransferase class-I	0.0085	1.62
CAG39306.1	SAR0279	6 kDa early secretory antigenic target ESAT-6 (EsxA)	0.0198	1.61
CAG40785.1	SAR1794	aminotransferase class-V protein	0.0099	1.61
CAG40374.1	SAR1376	4-oxalocrotonate tautomerase _	0.0014	1.61
CAG40867.1	SAR1877	2-succinylbenzoate--CoA ligase; OSB-CoA synthetase	0.0006	1.61
CAG41010.1	SAR2025	putative ABC transporter ATP-binding protein	0.0136	1.61
CAG40421.1	SAR1424	dihydrolipoyllysine-residue succinyltransferase	0.0033	1.61
CAG39207.1	SAR0180	putative non-ribosomal peptide synthetase	0.0272	1.60
CAG40095.1	SAR1093	YlbF family regulator; Cell fate regulator YlbF, YheA/YmcA/DUF963 family (controls sporulation, competence, biofilm development)	0.0236	1.59
CAG39630.1	SAR0611	HD domain-containing protein , putative phosphohydrolase	0.0013	1.59
CAG41208.1	SAR2227	DNA starvation/stationary phase protection protein ; putative non-heme iron-containing ferritin	0.0182	1.59
CAG39934.1	SAR0928	helicase-exonuclease AddAB subunit AddB	0.0014	1.58
CAG41686.1	SAR2708	Esterase family protein	0.0213	1.58
CAG41549.1	SAR2568	hypothetical protein SAR2568	0.0226	1.57
CAG40765.1	SAR1774	citrate synthase II	0.0305	1.57
CAG41210.1	SAR2229	EVE domain-containing protein	0.0055	1.57
CAG41165.1	SAR2184	putative exported protein	0.0062	1.57
CAG39926.1	SAR0920	Glu/Leu/Phe/Val dehydrogenase	0.0047	1.56
CAG39908.1	SAR0902	conserved hypothetical protein; Fe-S cluster assembly iron-binding protein IscA [Posttranslational modification, protein turnover IscA	0.0060	1.56
CAG40933.1	SAR1946	tRNA (uridine(34)/cytosine(34)/5-carboxymethylaminomethyluridine(34)-2'-O)-methyltransferase TrmL	0.0072	1.56
CAG40361.1	SAR1363	acyl-CoA thioesterase	0.0027	1.56
CAG41386.1	SAR2404	HAD family hydrolase; haloacid dehalogenase	0.0048	1.55
CAG39869.1	SAR0860	A Chain A, 3-dehydroquinate Dehydratase	0.0009	1.55
CAG39641.1	SAR0623	hypothetical protein	0.0451	1.55
CAG41014.1	SAR2028	Aminotransferase class I/II-fold pyridoxal phosphate-dependent enzyme	0.0212	1.55
CAG39670.1	SAR0653	ABC transporter ATP-binding protein;	0.0101	1.55
CAG40422.1	SAR1425	2-oxoglutarate dehydrogenase E1 component (EC 1.2.4.2)	0.0072	1.55
CAG39549.1	SAR0527	Arginine kinase	0.0002	1.54
CAG40685.1	SAR1693	putative O-methyltransferase	0.0008	1.54
CAG41285.1	SAR2304	Energy-coupling factor transporter ATP-binding protein EcfA2; Short=ECF transporter A component EcfA2	0.0007	1.54
CAG40124.1	SAR1121	putative succinate dehydrogenase flavoprotein subunit	0.0008	1.54
CAG39190.1	SAR0163	sugar transferase; capsule biosynthesis	0.0268	1.54
CAG41375.1	SAR2393	Formate dehydrogenase	0.0003	1.54
CAG40807.1	SAR1816	putative membrane protein	0.0028	1.53
CAG40806.1	SAR1815	DAHP synthetase-chorismate mutase	0.0000	1.53
CAG39510.1	SAR0488	tRNA1(Val) (adenine(37)-N6)-methyltransferase	0.0049	1.52
CAG40578.1	SAR1583	AraC family transcriptional regulator	0.0102	1.52
CAG39368.1	SAR0345	Conserved hypothetical protein	0.0419	1.52
CAG39851.1	SAR0842	MSCRAMM family adhesin clumping factor ClfA	0.0134	1.52
CAG40794.1	SAR1803	PTS system IIBC component; PTS system, N-acetylglucosamine-specific IIBC component	0.0015	1.52
CAG41084.1	SAR2101	putative exonuclease/ATP-dependent helicase - DNA Pol III alpha subunit	0.0025	1.52
CAG40439.1	SAR1442	conserved hypothetical protein	0.0008	1.51
CAG39141.1	SAR0115	HTH-type transcriptional regulator SarS	0.0082	1.51
CAG39419.1	SAR0396	putative lipoprotein	0.0108	1.51
CAG40953.1	SAR1966	hypothetical protein	0.0005	1.51
CAG40199.1	SAR1197	ribosome small subunit-dependent GTPase A	0.0284	1.51
CAG41396.1	SAR2416	imidazolonepropionase	0.0028	1.51
CAG40233.1	SAR1231	GTP-sensing pleiotropic transcriptional regulator CodY	0.0163	-1.52
CAG39800.1	SAR0790	siderophore ABC transporter substrate-binding protein	0.0022	-1.52
CAG41305.1	SAR2324	50S ribosomal protein L24	0.0056	-1.52
CAG39593.1	SAR0572	YojF family protein	0.0478	-1.53
CAG39043.1	SAR0015	50S ribosomal protein L9	0.0074	-1.53
CAG41661.1	SAR2684	Fructose-bisphosphate aldolase class 1	0.0056	-1.53
CAG41282.1	SAR2301	50S ribosomal protein L13	0.0029	-1.55
CAG41039.1	SAR2054	hypothetical phage protein	0.0119	-1.55
CAG40860.1	SAR1869	putative exported protein; nuclease-related domain	0.0129	-1.55
CAG41581.1	SAR2601	acetyltransferase (GNAT) family protein	0.0134	-1.56
CAG41548.1	SAR2567	SDR family oxidoreductase	0.0162	-1.56
CAG40210.1	SAR1208	acyl carrier protein	0.0145	-1.57
CAG39565.1	SAR0544	50S ribosomal protein L10	0.0035	-1.59
CAG41504.1	SAR2523	membrane protein	0.0062	-1.60
CAG40696.1	SAR1705	conserved hypothetical protein	0.0007	-1.61
CAG40344.1	SAR1345	50S ribosomal protein L33	0.0205	-1.61
CAG40775.1	SAR1784	universal stress protein	0.0000	-1.62
CAG40432.1	SAR1435	PTS system glucose-specific EIIA component;	<0.000001	-1.62
CAG41542.1	SAR2561	Alkylhydroperoxidase family enzyme, contains CxxC motif	0.0157	-1.63
CAG39740.1	SAR0730	putative lipoprotein	0.0002	-1.63
CAG40996.1	SAR2011	isochorismatase family protein	0.0006	-1.64
CAG40346.1	SAR1347	GMP reductase (EC 1.7.1.7)	0.0000	-1.66

CAG39678.1	SAR0661	putative dihydroxyacetone kinase subunit L	0.0069	-1.67
CAG39261.1	SAR0234	L-lactate dehydrogenase	<0.000001	-1.69
CAG39609.1	SAR0589	putative amino acid permease	0.0016	-1.70
CAG41251.1	SAR2273	Asp23/Gls24 family envelope stress response protein	0.0071	-1.70
CAG39763.1	SAR0753	PTS transporter subunit EIIA; PTS transport system, fructose-specific IIABC component	0.0042	-1.74
CAG40411.1	SAR1414	cold-shock protein CspA	0.0034	-1.78
CAG39762.1	SAR0752	1-phosphofructokinase	0.0010	-1.80
CAG41560.1	SAR2580	fibronectin-binding protein precursor FnbA	0.0009	-1.81
CAG39838.1	SAR0829	A Chain A, Phosphoglycerate Kinase	0.0000	-1.86
CAG39837.1	SAR0828	P Chain P, Glyceraldehyde-3-phosphate Dehydrogenase 1	0.0022	-1.86
CAG39744.1	SAR0734	conserved hypothetical protein	0.0056	-1.90
CAG41340.1	SAR2359	putative molybdenum cofactor biosynthesis protein B	0.0038	-1.91
CAG41309.1	SAR2328	50S ribosomal protein L16	0.0239	-1.91
CAG41388.1	SAR2407	hypothetical protein SAR2407	0.0018	-1.96
CAG41295.1	SAR2314	adenylate kinase	0.0211	-1.98
CAG41312.1	SAR2331	30S ribosomal protein S19	0.0249	-1.99
CAG43128.1	SAR1422	hypothetical protein	0.0138	-1.99
CAG41298.1	SAR2317	50S ribosomal protein L30	0.0135	-1.99
CAG40746.1	SAR1755	trigger factor (prolyl isomerase)	0.0022	-2.00
CAG40176.1	SAR1174	pyrimidine operon regulatory protein	0.0465	-2.09
CAG40788.1	SAR1797	30S ribosomal protein S4	0.0025	-2.10
Q6GGW4.1	SAR1457	Cell cycle protein GpsB; Guiding PBP1-shuttling protein	0.0029	-2.19
CAG41031.1	SAR2046	hypothetical phage protein ;phage major tail protein, phi13 family; TIGR01603	0.0048	-2.25
CAG39761.1	SAR0751	DeoR/GlpR transcriptional regulator	0.0003	-2.33
CAG40849.1	SAR1858	hypothetical protein SAR1858	0.0013	-2.38
CAG39936.1	SAR0930	Uncharacterized protein; putative fumarylacetoacetate (FAA) hydrolase family protein	0.0101	-2.40
CAG40102.1	SAR1100	conserved hypothetical protein	0.0057	-2.46
CAG41162.1	SAR2182	Bifunctional hydroxymethylpyrimidine kinase/phosphomethylpyrimidine kinase	0.0219	-2.88
CAG40251.1	SAR1249	30S ribosomal protein S15	0.0054	-3.36
CAG40686.1	SAR1694	UPF0473 protein SAR1694	0.0018	-3.42
CAG40434.1	SAR1437	Peptide methionine sulfoxide reductase MsrA 2	0.0000	-3.73
CAG40474.1	SAR1476	3-dehydroquinate synthase	0.0033	-4.68
CAG39598.1	SAR0577	proline/betaine transporter	0.0044	-6.71
CAG39699.1	SAR0683	LysR family transcriptional regulator	0.0003	-7.90
CAG41171.1	SAR2190	ATP synthase epsilon chain; ATP synthase F1 sector epsilon subunit	<0.000001	-10.12

Supplemental Table 3. Secretomics data from spaceflight (FLT) vs. ground control (GC) cultures

PROTEIN ID	GENE ID	FUNCTION	P VALUE	RATIO (FL/GC)
CAG41617.1	SAR2639	CopZ putative heavy-metal-associated protein	< 0.00010	18.19
CAG41689.1	SAR2711	carbamate kinase	0.0014	14.75
CAG40025.1	SAR1021	Cysteine protease precursor; SspB	< 0.00010	13.07
CAG39500.1	SAR0478	conserved hypothetical protein	0.019	10.06
CAG40808.1	SAR1817	putative exported protein	0.00012	8.46
CAG39855.1	SAR0847	thermonuclease precursor	< 0.00010	7.72
CAG39306.1	SAR0279	6 kDa early secretory antigenic target ESAT-6 (EsxA)	0.0004	7.50
CAG39620.1	SAR0600	pyridine nucleotide-disulphide oxidoreductase protein	0.028	7.25
CAG40986.1	SAR2001	staphopain protease	< 0.00010	7.18
CAG40026.1	SAR1022	Glutamyl endopeptidase precursor; V8 Protease; SspA	< 0.00010	6.95
CAG39832.1	SAR0823	putative ATP-dependent Clp protease proteolytic subunit	0.041	6.70
CAG40105.1	SAR1103	iron-regulated heme-iron binding protein	0.049	5.69
CAG40969.1	SAR1983	Mur ligase family protein	0.013	5.40
CAG41441.1	SAR2459	putative zinc-binding dehydrogenase	0.036	5.30
CAG41488.1	SAR2508	IgG-binding protein	< 0.00010	4.94
CAG40632.1	SAR1637	conserved hypothetical protein	0.0058	4.85
CAG39999.1	SAR0994	putative 5'-nucleotidase	0.0072	4.83
CAG41281.1	SAR2300	30S ribosomal protein S9 RpsI	0.048	4.37
CAG41358.1	SAR2377	Urease accessory protein UreG	0.028	4.37
CAG39807.1	SAR0797	putative peptidase T	0.0036	4.36
CAG41354.1	SAR2373	Urease beta subunit (EC 3.5.1.5)	0.027	4.35
CAG39732.1	SAR0721	Multicopper oxidase protein	0.0026	4.08
CAG39485.1	SAR0464	putative exported protein	0.019	3.85
CAG40130.1	SAR1127	Hypothetical protein, similarity with fibrinogen-binding protein Efb	0.042	3.78
CAG41109.1	SAR2128	sucrose-6-phosphate hydrolase	0.03	3.26
CAG40066.1	SAR1064	conserved hypothetical protein	0.028	3.24
CAG41467.1	SAR2486	nitrate reductase alpha chain	0.015	3.13
CAG40813.1	SAR1822	putative thioredoxin	0.0034	3.08
CAG41692.1	SAR2714	Arginine deiminase ArcA	< 0.00010	3.04
CAG39368.1	SAR0345	Conserved hypothetical protein	0.017	2.94
CAG41340.1	SAR2359	putative molybdenum cofactor biosynthesis protein B	0.044	2.91
CAG40484.1	SAR1486	cytidylate kinase	0.0075	2.90
CAG39613.1	SAR0593	conserved hypothetical protein	0.036	2.84
CAG41189.1	SAR2208	putative 50S ribosomal protein L31	0.02	2.72
CAG41614.1	SAR2635	Putative acetyltransferase SAR2635	0.022	2.69
CAG41687.1	SAR2709	Chain A, Clumping Factor B (ClfB)	0.0033	2.67
CAG40268.1	SAR1266	conserved hypothetical protein	0.048	2.43
CAG39514.1	SAR0492	putative TatD related DNase	0.021	2.31
CAG41691.1	SAR2713	putative ornithine carbamoyltransferase	0.00042	2.30
CAG41694.1	SAR2716	Zinc metalloproteinase aureolysin; Aur	0.0076	2.19
CAG40463.1	SAR1465	putative asparaginyl-tRNA synthetase	0.042	2.16
CAG40923.1	SAR1936	conserved hypothetical protein	0.013	2.05
CAG41014.1	SAR2028	Aminotransferase class I/II-fold pyridoxal phosphate-dependent enzyme	0.017	2.03
CAG39880.1	SAR0874	conserved hypothetical protein YjbI	0.0069	2.02
CAG39845.1	SAR0836	putative ribonuclease R	0.012	1.99
CAG39950.1	SAR0944	putative exported protein	0.012	1.87
CAG39817.1	SAR0807	preprotein translocase SecA subunit	0.015	1.84
CAG41355.1	SAR2374	Urease alpha subunit (EC 3.5.1.5)	0.0056	1.82
CAG41172.1	SAR2191	F0F1 ATP synthase subunit beta	0.0044	1.81
CAG39035.1	SAR0007	ADP-dependent (S)-NAD(P)H-hydrate dehydratase (EC 4.2.1.136)	0.015	1.80
CAG39640.1	SAR0622	putative exported protein	< 0.00010	1.78
CAG40219.1	SAR1217	50S ribosomal protein L19	0.00058	1.71
CAG40788.1	SAR1797	30S ribosomal protein S4	0.0019	1.69
CAG40121.1	SAR1118	thioredoxin	0.00039	1.68
CAG39279.1	SAR0253	putative zinc-binding dehydrogenase	0.002	1.66
CAG40861.1	SAR1870	Methionine adenosyltransferase	0.0094	1.61
CAG40970.1	SAR1984	Bacterial non-heme ferritin (EC 1.16.3.2)	0.0057	1.58
CAG40797.1	SAR1806	tyrosyl-tRNA synthetase	0.0027	1.58
CAG40213.1	SAR1211	putative cell division protein	0.047	1.52
CAG40067.1	SAR1065	putative polypeptide deformylase 2	0.0024	1.51
CAG40228.1	SAR1226	DNA topoisomerase I	0.012	-1.50
CAG41039.1	SAR2054	hypothetical phage protein	0.0019	-1.53
CAG39177.1	SAR0150	putative aldehyde-alcohol dehydrogenase	0.026	-1.54
CAG39990.1	SAR0985	conserved hypothetical protein	0.03	-1.56
CAG41486.1	SAR2506	phosphoglycerate mutase	0.0092	-1.56
CAG39576.1	SAR0555	putative 2-amino-3-ketobutyrate coenzyme A ligase	0.00077	-1.56
CAG39540.1	SAR0518	lysyl-tRNA synthetase	0.001	-1.57
CAG39643.1	SAR0625	staphylococcal accessory regulator A	0.0015	-1.59
CAG39658.1	SAR0641	ABC transporter extracellular binding protein	0.00091	-1.66
CAG39771.1	SAR0761	putative lipoprotein	0.0028	-1.66
CAG40701.1	SAR1710	aspartyl-tRNA synthetase	0.0013	-1.68
CAG40951.1	SAR1964	putative transglycosylase	0.0013	-1.71
CAG40163.1	SAR1161	Chain A, Cell Division Protein Ftsa	0.0038	-1.72

CAG39034.1	SAR0006	DNA gyrase subunit A	0.0027	-1.74
CAG40715.1	SAR1724	Spo0B-associated GTP-binding protein	0.014	-1.83
CAG41770.1	SAR2798	probable tRNA modification GTPase	0.048	-1.83
CAG40925.1	SAR1938	putative DNA-binding protein	0.00029	-1.84
CAG40179.1	SAR1177	putative dihydroorotase	0.008	-1.85
CAG39897.1	SAR0891	haloacid dehalogenase-like hydrolase	0.0065	-1.88
CAG41598.1	SAR2619	thiamine pyrophosphate enzyme	0.043	-1.88
CAG39167.1	SAR0140	deoxyribose-phosphate aldolase	0.0096	-1.89
CAG41228.1	SAR2247	putative mannitol-1-phosphate 5-dehydrogenase	0.0033	-1.91
CAG40090.1	SAR1088	putative pyruvate carboxylase	0.0096	-1.94
CAG39745.1	SAR0735	putative exported protein	0.018	-2.00
CAG41168.1	SAR2187	FabZ putative hydroxymyristoyl-(acyl carrier protein) dehydratase	0.02	-2.03
CAG40181.1	SAR1179	putative carbamoyl-phosphate synthase, pyrimidine-specific, large chain CarB	0.0091	-2.04
CAG40189.1	SAR1187	putative flavoprotein	0.0021	-2.10
CAG40981.1	SAR1996	DNA ligase	0.015	-2.11
CAG40154.1	SAR1152	acetyltransferase (GNAT) family protein	0.021	-2.14
CAG41235.1	SAR2256	conserved hypothetical protein	0.033	-2.37
CAG39119.1	SAR0092	putative hydratase	0.022	-2.39
CAG39412.1	SAR0390	putative lipoprotein	< 0.00010	-2.49
CAG40592.1	SAR1597	putative DNA repair protein	0.046	-2.50
CAG40248.1	SAR1246	putative ribosome-binding factor A	0.017	-2.53
CAG39182.1	SAR0155	capsular polysaccharide synthesis enzyme	0.002	-2.56
CAG39029.1	SAR0001	chromosomal replication initiator protein DnaA	0.0024	-2.82
CAG40952.1	SAR1965	ThiJ/PfpI family protein	0.039	-2.85
CAG39684.1	SAR0667	putative acetyltransferase	0.027	-2.87
CAG40624.1	SAR1629	penicillin-binding protein PBP2B	0.047	-2.88
CAG39374.1	SAR0351	acetyl-CoA acetyltransferase	0.00062	-3.02
CAG40002.1	SAR0997	putative lipoate-protein ligase A	0.0082	-3.22
CAG40570.1	SAR1574	iron uptake regulatory protein	0.00095	-3.56
CAG40710.1	SAR1719	queuine tRNA-ribosyltransferase	0.028	-3.92
CAG40175.1	SAR1173	putative RNA pseudouridylate synthase	< 0.00010	-5.73
CAG40593.1	SAR1598	RecName: Full=Arginine repressor, ArgR	< 0.00010	-8.51

Supplemental Table 4. STRING functional enrichment analysis of RNA-seq, proteomics, and secretomics data

Category	Term ID	Enriched Functional Category	FDR
		RNASeq	
GO Process	GO:0016051	Carbohydrate biosynthetic process	0.026
GO Function	GO:0016151	Nickel cation binding	0.0011
STRING clusters	CL:4039	Mixed, incl. Tuberculosis, and serine-type D-Ala-D-Ala carboxypeptidase activity	0.00069
STRING clusters	CL:3657	Polysaccharide biosynthetic process, and O-Antigen nucleotide sugar biosynthesis	0.0022
STRING clusters	CL:2646	Quorum sensing, and phosphorelay signal transduction system	0.005
STRING clusters	CL:724	Carbohydrate metabolic process, and Phosphotransferase system (PTS)	0.005
STRING clusters	CL:3311	Mixed, incl. divalent inorganic cation transmembrane transporter activity, and Arsenical resistance	0.0066
STRING clusters	CL:4027	Mixed, incl. Heme, and Virulence	0.0066
STRING clusters	CL:839	Starch and sucrose metabolism	0.0066
STRING clusters	CL:939	D-ribose metabolic process, and carbohydrate transmembrane transport	0.0365
STRING clusters	CL:726	Carbohydrate metabolic process, and Phosphotransferase system (PTS)	0.0403
		Proteomics	
GO Process	GO:0008152	Metabolic process	0.018
GO Function	GO:0005488	Binding	0.0023
STRING clusters	CL:4661	Nickel cation binding	0.0179
		Secretomics	
GO Process	GO:0008152	Metabolic process	9.99E-05
GO Process	GO:0044237	Cellular metabolic process	0.0057
GO Process	GO:0034641	Cellular nitrogen compound metabolic process	0.0149
GO Process	GO:0043170	Macromolecule metabolic process	0.0224
GO Process	GO:0044281	Small molecule metabolic process	0.0498
GO Function	GO:0043167	Ion binding	2.88E-05
GO Function	GO:0005488	Binding	0.00018
GO Function	GO:0003824	Catalytic activity	0.00037
GO Function	GO:0043169	Cation binding	0.00051
GO Function	GO:0046872	Metal ion binding	0.00081
GO Function	GO:0043168	Anion binding	0.0098
KEGG	sao00220	Arginine biosynthesis	0.0301

Supplemental Table 5. Cellular metabolite data from spaceflight vs. ground control cultures

METABOLITE	RATIO (FL/GC)	P-VALUE	FDR
isothreonic acid	12.31	4.12E-12	7.31E-10
ribonic acid	7.70	2.24E-05	1.75E-04
methanolphosphate	7.38	3.68E-09	2.62E-07
n-acetyl-d-hexosamine	6.08	2.38E-08	1.16E-06
O-acetylserine	5.31	2.85E-06	3.38E-05
ornithine	4.90	2.47E-07	5.21E-06
phosphoenolpyruvate	4.88	1.70E-07	4.12E-06
xylitol	3.94	2.39E-08	1.16E-06
ribitol	3.74	4.43E-06	4.76E-05
3-phosphoglycerate	3.57	2.10E-05	1.67E-04
adenosine-5-monophosphate	3.52	1.55E-05	1.34E-04
glucose-6-phosphate	3.33	1.85E-03	7.38E-03
putrescine	3.20	3.63E-05	2.55E-04
glycerol-alpha-phosphate	3.12	2.09E-05	1.67E-04
gluconic acid	3.11	6.35E-03	1.77E-02
methionine sulfoxide	2.98	8.11E-08	2.27E-06
ribose	2.88	7.97E-07	1.12E-05
glyceric acid	2.88	3.05E-03	1.04E-02
thymine	2.86	7.06E-07	1.07E-05
citramalic acid	2.83	2.05E-06	2.61E-05
glycerol-3-galactoside	2.83	1.53E-05	1.34E-04
arabinose	2.70	5.97E-07	9.36E-06
lactulose	2.70	2.69E-04	1.38E-03
galactonic acid	2.58	2.21E-02	4.41E-02
phenylethylamine	2.47	4.84E-03	1.44E-02
glycolic acid	2.41	1.42E-09	1.51E-07
homoserine	2.25	4.20E-04	2.07E-03
cysteine	2.21	4.64E-03	1.41E-02
alpha-ketoglutarate	2.20	4.86E-05	3.20E-04
3-phenyllactic acid	2.19	5.32E-07	8.87E-06
N-carbamoylaspartate	2.17	8.04E-05	4.98E-04
guanine	2.16	1.19E-04	6.92E-04
lyxitol	2.16	1.27E-05	1.15E-04
pyrophosphate	2.09	1.01E-02	2.44E-02
arabitol	2.07	2.62E-05	1.97E-04
glutamine	2.06	3.70E-05	2.56E-04
beta-glycerolphosphate	2.03	2.27E-02	4.48E-02
3-hydroxypropionic acid	2.03	4.42E-09	2.62E-07
adenine	2.00	3.99E-05	2.69E-04
aconitic acid	1.96	1.24E-02	2.86E-02

4-aminobutyric acid	1.91	6.04E-04	2.88E-03
threonic acid	1.86	5.50E-05	3.57E-04
N-acetylputrescine	1.85	5.07E-04	2.46E-03
tagatose	1.84	7.36E-03	1.97E-02
2-hydroxybutanoic acid	1.81	2.60E-02	4.96E-02
methylhexose nist	1.78	1.01E-02	2.44E-02
fumaric acid	1.72	6.10E-03	1.72E-02
guanidinosuccinate	1.71	4.29E-03	1.33E-02
phosphoethanolamine	1.69	2.82E-02	5.22E-02
saccharopine	1.69	1.22E-02	2.83E-02
hypoxanthine	1.67	3.57E-03	1.17E-02
maleimide	1.67	2.41E-03	8.81E-03
isothreitol	1.67	4.26E-02	7.32E-02
glycocyamine	1.66	1.04E-02	2.49E-02
parabanic acid NIST	1.65	1.34E-03	5.60E-03
oxoproline	1.63	3.84E-03	1.23E-02
proline	1.62	5.74E-03	1.66E-02
lactic acid	1.60	3.19E-03	1.08E-02
tyrosine	1.58	7.15E-03	1.94E-02
phenylalanine	1.58	6.13E-03	1.72E-02
asparagine	1.56	5.77E-03	1.66E-02
cytidine	1.52	3.79E-02	6.65E-02
glycine	0.45	3.02E-05	2.21E-04
dihydroxyacetone	0.43	3.74E-03	1.22E-02
sucrose	0.42	4.95E-02	8.27E-02
xanthine	0.33	1.63E-03	6.57E-03
fructose	0.12	6.01E-05	3.81E-04
methionine	0.04	1.17E-11	1.55E-09

Supplemental Table 6. Supernatant metabolite data from spaceflight vs. ground control cultures

METABOLITE	RATIO (FL/GC)	P-VALUE	FDR
O-acetylserine	11.45	3.79E-08	1.35E-06
isothreonic acid	7.92	8.23E-12	7.31E-10
ribonic acid	5.36	1.61E-13	2.14E-11
ornithine	3.10	6.50E-05	5.08E-04
n-acetyl-d-hexosamine	2.89	2.02E-05	1.96E-04
thymine	2.81	4.58E-02	1.38E-01
glycerol-3-galactoside	2.66	4.14E-05	3.56E-04
alpha-ketoglutarate	2.65	1.48E-04	9.97E-04
3-phenyllactic acid	2.59	3.24E-05	2.89E-04
beta-glycerolphosphate	2.36	4.55E-04	2.70E-03
putrescine	2.35	4.86E-07	1.04E-05
citramalic acid	2.31	7.32E-05	5.57E-04
gluconic acid	2.26	1.88E-05	1.86E-04
methionine sulfoxide	2.25	3.67E-09	1.96E-07
xylitol	2.24	4.72E-04	2.74E-03
oxalic acid	2.11	1.13E-03	5.45E-03
glycerol-alpha-phosphate	1.93	9.79E-03	3.70E-02
glycolic acid	1.90	6.21E-06	8.05E-05
phosphoenolpyruvate	1.87	1.54E-05	1.67E-04
pyrophosphate	1.85	3.36E-02	1.09E-01
N-carbamoylaspartate	1.79	4.48E-05	3.79E-04
lyxitol	1.73	7.96E-04	4.24E-03
glyceric acid	1.72	2.27E-05	2.16E-04
2-aminobutyric acid	1.69	1.75E-02	6.13E-02
galactinol	1.67	1.99E-03	9.09E-03
threonic acid	1.65	2.12E-06	3.32E-05
palmitic acid	1.64	4.80E-02	1.42E-01
lactic acid	1.60	2.23E-02	7.46E-02
glutamine	1.54	1.47E-02	5.36E-02
homoserine	1.54	7.76E-03	3.02E-02
3-hydroxypropionic acid	1.52	4.23E-03	1.77E-02
glucose	0.49	5.12E-05	4.07E-04
malic acid	0.48	1.89E-06	3.14E-05
3,6-anhydro-D-galactose	0.48	1.80E-05	1.86E-04
digitoxose	0.46	1.64E-08	6.73E-07
guanosine	0.36	3.25E-05	2.89E-04
trehalose	0.36	3.09E-03	1.34E-02
glycine	0.34	2.47E-05	2.31E-04
aminomalonate	0.33	6.53E-06	8.09E-05
inosine	0.32	7.82E-05	5.79E-04

dihydroxyacetone	0.30	8.37E-05	6.03E-04
adenosine	0.27	1.47E-03	6.83E-03
sucrose	0.17	4.74E-08	1.58E-06
xanthine	0.17	9.49E-04	4.91E-03
fructose	0.08	3.01E-06	4.33E-05
methionine	0.01	4.44E-19	2.37E-16

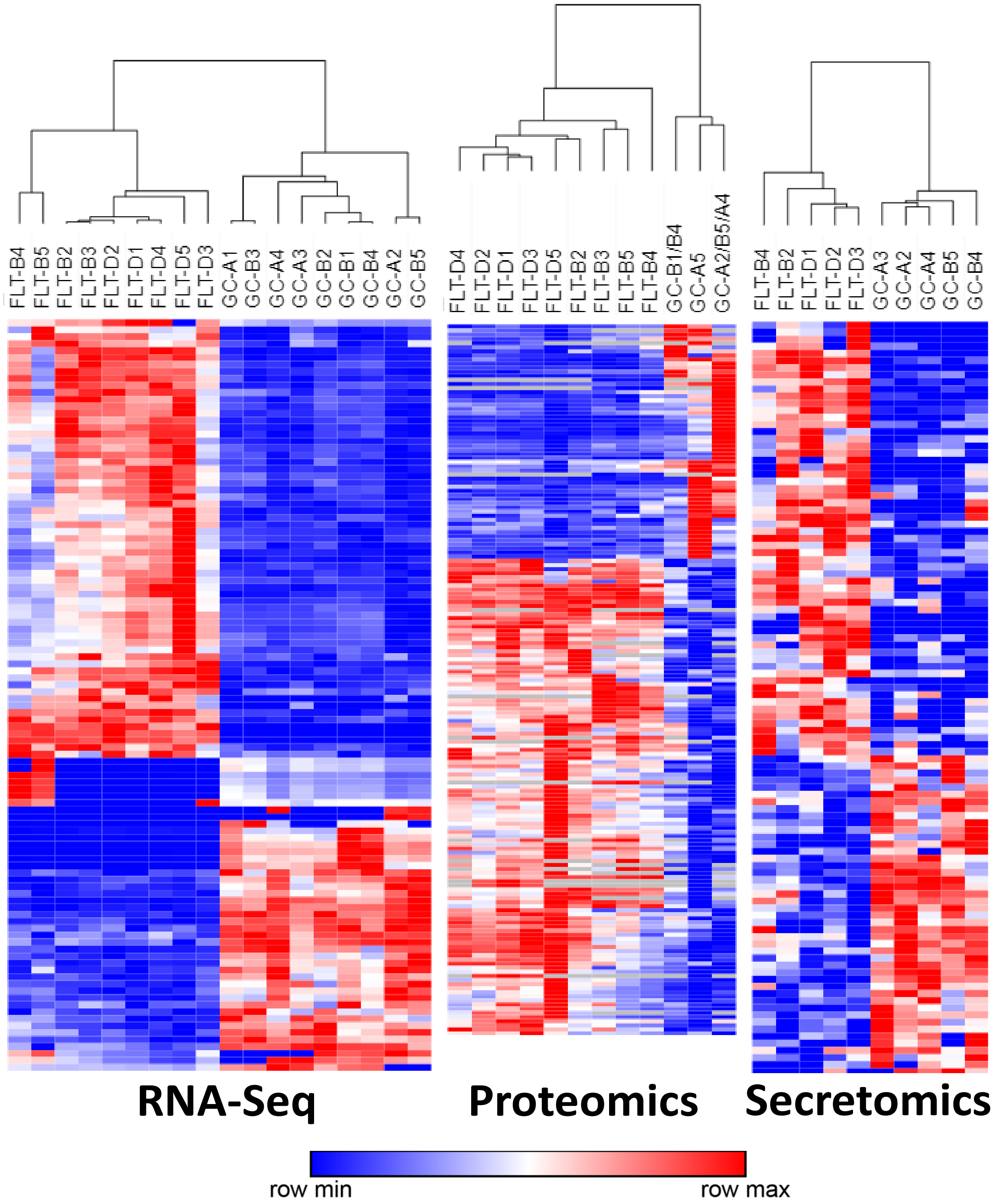

Supplemental Figure 1. Heat maps of statistically-significant gene/protein changes between BRIC-23 flight (FLT) and ground control (GC) cultures. Hierarchical clustering using 1-Pearson correlation on rows and columns, using Morpheus software (https://software.broadinstitute.org/morpheus/).

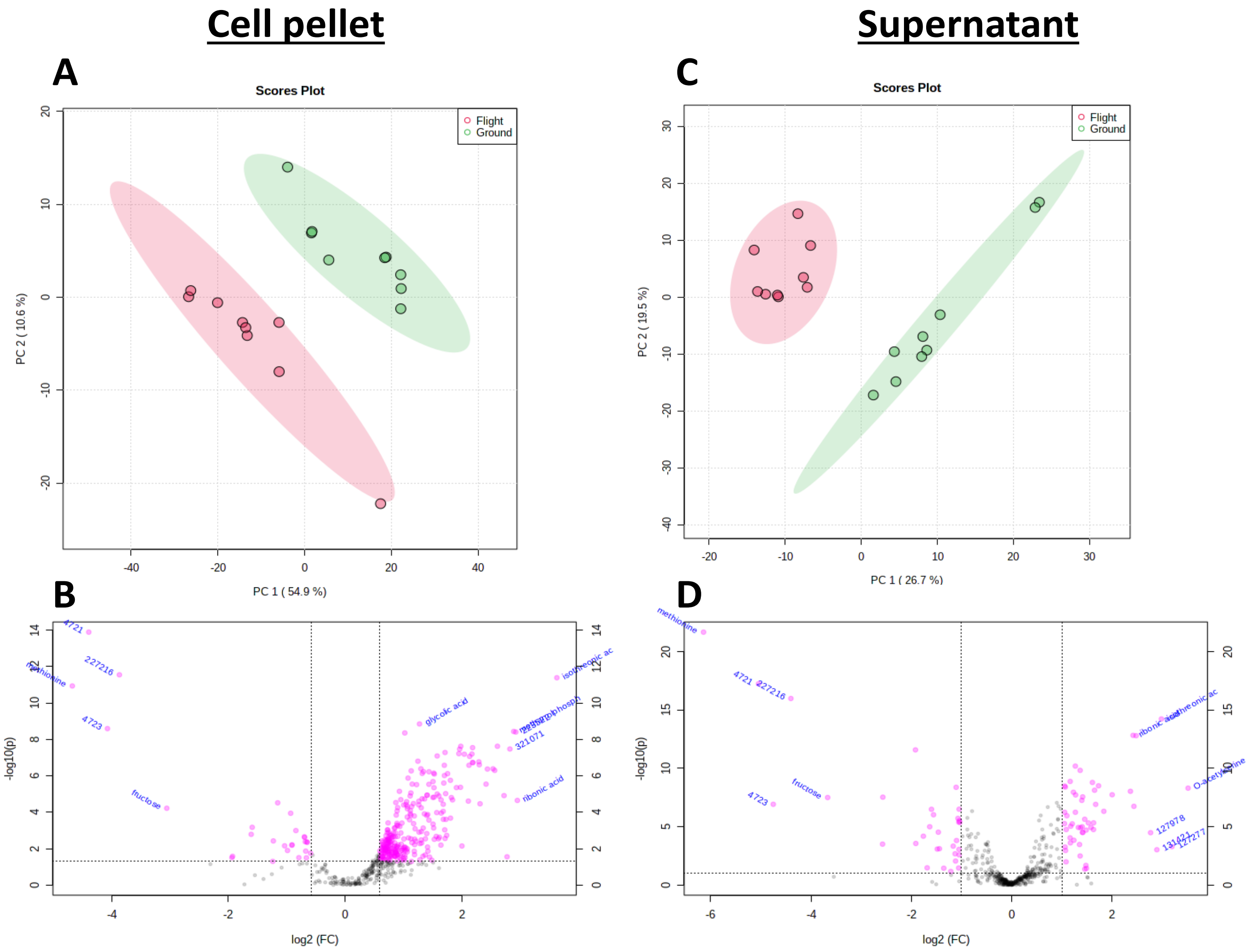

Supplemental Figure 2. PCA (A and B) and volcano plots (C and D) of BRIC-23 untargeted metabolomics data.
