# Supplementary material for: Altered quorum sensing and physiology of Staphylococcus aureus during spaceflight detected by multi-omics data analysis
Source: NPJ Microgravity. 2024 Jan 8;10:2. doi: 10.1038/s41526-023-00343-7 (PMC10774393; doi:10.1038/s41526-023-00343-7)
Supplement: Supplementary file 2 — Reporting Summary [file 41526_2023_343_MOESM2_ESM.pdf]

nature portfolio

Corresponding author(s): Kelly Rice

Last updated by author(s): Oct 26, 2023

Reporting Summary

Nature Portfolio wishes to improve the reproducibility of the work that we publish. This form provides structure for consistency and transparency in reporting. For further information on Nature Portfolio policies, see our Editorial Policies and the Editorial Policy Checklist.

Statistics

For all statistical analyses, confirm that the following items are present in the figure legend, table legend, main text, or Methods section.

n/a	Confirmed	
☐	☒	The exact sample size (*n*) for each experimental group/condition, given as a discrete number and unit of measurement
☐	☒	A statement on whether measurements were taken from distinct samples or whether the same sample was measured repeatedly
☐	☒	The statistical test(s) used AND whether they are one- or two-sided<br>*Only common tests should be described solely by name; describe more complex techniques in the Methods section.*
☐	☒	A description of all covariates tested
☐	☒	A description of any assumptions or corrections, such as tests of normality and adjustment for multiple comparisons
☐	☒	A full description of the statistical parameters including central tendency (e.g. means) or other basic estimates (e.g. regression coefficient) AND variation (e.g. standard deviation) or associated estimates of uncertainty (e.g. confidence intervals)
☐	☒	For null hypothesis testing, the test statistic (e.g. *F*, *t*, *r*) with confidence intervals, effect sizes, degrees of freedom and *P* value noted<br>*Give P values as exact values whenever suitable.*
☒	☐	For Bayesian analysis, information on the choice of priors and Markov chain Monte Carlo settings
☒	☐	For hierarchical and complex designs, identification of the appropriate level for tests and full reporting of outcomes
☒	☐	Estimates of effect sizes (e.g. Cohen's *d*, Pearson's *r*), indicating how they were calculated

Our web collection on statistics for biologists contains articles on many of the points above.

Software and code

Policy information about availability of computer code

Data collection	No software was used
Data analysis	Statistical analysis was performed using Microsoft Excel, Graphpad Prism 9, or Scaffold software version 4.11.0. Functional categories of genes/proteins identified by RNA-Seq, proteomics, and secretomics as being statistically significant (> 2-fold change in expression for RNA-Seq, and >1.5-fold change for proteomics and secretomics) were assigned using manual curation based on gene annotation and/or predicted function using the following databases: Aureowiki, Uniprot, PATRIC and Biocyc. Hierarchical clustering and heat map generation of statistically significant DE data was performed using 1-Pearson correlation on rows and columns, using Morpheus default settings (https://software.broadinstitute.org/morpheus). Venn diagram of overlapping and unique DE genes/proteins identified by RNA-Seq, proteomics, and secretomics as being statistically significant with > 2-fold change in expression for RNA-Seq and > 1.5-fold change for proteomics and secretomics, was generated using OmicsBox (BioBam, Valencia, Spain). Principal Components Analysis (PCA) of RNA-Seq, proteomics, and secretomics data was performed using ClustVis. PCA plots for metabolomics data were generated with Rscript chemometrics.R within the MetaboAnalyst program. RNA-Seq, cellular proteomics, and/or secretomics DE data were analyzed using Paint-omics version 3 and STRING version 11.5 to detect enriched KEGG pathways and genes/proteins, respectively.

For manuscripts utilizing custom algorithms or software that are central to the research but not yet described in published literature, software must be made available to editors and reviewers. We strongly encourage code deposition in a community repository (e.g. GitHub). See the Nature Portfolio guidelines for submitting code & software for further information.

Data

Policy information about availability of data

All manuscripts must include a data availability statement. This statement should provide the following information, where applicable:

- Accession codes, unique identifiers, or web links for publicly available datasets
- A description of any restrictions on data availability
- For clinical datasets or third party data, please ensure that the statement adheres to our policy

All BRIC-23 -omics datasets and corresponding metadata can be accessed through NASA OSDR (for RNASeq, proteomics, and metabolomics: DOI: 10.26030/ga0p-2817, for secretomics: DOI: 10.26030/rztr-e997). All other data presented in this manuscript is available as figures/tables, or supplementary figures/tables.

Research involving human participants, their data, or biological material

Policy information about studies with human participants or human data. See also policy information about sex, gender (identity/presentation), and sexual orientation and race, ethnicity and racism.

Reporting on sex and gender	Not applicable
Reporting on race, ethnicity, or other socially relevant groupings	Not applicable
Population characteristics	Not applicable
Recruitment	Not applicable
Ethics oversight	Not applicable

Note that full information on the approval of the study protocol must also be provided in the manuscript.

Field-specific reporting

Please select the one below that is the best fit for your research. If you are not sure, read the appropriate sections before making your selection.

☒ Life sciences ☐ Behavioural & social sciences ☐ Ecological, evolutionary & environmental sciences

For a reference copy of the document with all sections, see nature.com/documents/nr-reporting-summary-flat.pdf

Life sciences study design

All studies must disclose on these points even when the disclosure is negative.

Sample size	For RNASeq, n=9 FLT and n=9 GC; For proteomics n=9 FLT and n=3 GC (n=9 GCs were pooled into n=3 samples submitted for proteomics, due to sample concentrations being too low for some, per BRIC-23 metadata); For secretomics, n=5 FLT and n=5 GC; For metabolomics, n=9 FLT and n=9 GC; For total CFU recovered per plate, n=9 each for FLT and GC; For supernatant protein concentrations, n=9 each for FLT and GC.
Data exclusions	N/A
Replication	Within the experimental design, there were n=9 total biological samples each for FLT and GC. Given the nature of the experiment (spaceflight), this in itself was not repeated.
Randomization	N/A
Blinding	N/A

Reporting for specific materials, systems and methods

We require information from authors about some types of materials, experimental systems and methods used in many studies. Here, indicate whether each material, system or method listed is relevant to your study. If you are not sure if a list item applies to your research, read the appropriate section before selecting a response.

Materials & experimental systems

n/a	Involved in the study
☒	☐ Antibodies
☒	☐ Eukaryotic cell lines
☒	☐ Palaeontology and archaeology
☒	☐ Animals and other organisms
☒	☐ Clinical data
☒	☐ Dual use research of concern
☒	☐ Plants

Methods

n/a	Involved in the study
☒	☐ ChIP-seq
☒	☐ Flow cytometry
☒	☐ MRI-based neuroimaging

Plants

Seed stocks	not applicable
Novel plant genotypes	not applicable
Authentication	not applicable
